# An examination of former prisoners’ mental health problems before death by suicide over a 21-year period (2001–2021)

**DOI:** 10.1192/bjo.2025.71

**Published:** 2025-06-23

**Authors:** Alison Baird, Lana Bojanić, Saied Ibrahim, Jessica Kenworthy, Pauline Turnbull, Navneet Kapur, Louis Appleby, Jenny Shaw, Daniel Pratt

**Affiliations:** National Confidential Inquiry into Suicide and Safety in Mental Health, Centre for Mental Health and Safety, Division of Psychology and Mental Health, School of Health Sciences, Faculty of Biology, Medicine and Health, University of Manchester, Manchester, UK; School of Health, Science and Wellbeing, University of Staffordshire, Stoke-on-Trent Campus, UK; Division of Psychology and Mental Health, University of Manchester, Manchester, UK; Manchester Academic Health Science Centre, Manchester, UK; Greater Manchester Mental Health NHS Foundation Trust, Manchester, UK

**Keywords:** Suicide, prison, ex-prisoner, mental health patients, childhood abuse

## Abstract

**Background:**

Former prisoners are a vulnerable population, and suicide rates among this group are high, particularly following release from prison.

**Aims:**

To explore former prisoners’ engagement with mental health services before death by suicide, and to examine the demographics, clinical history and clinical care of this patient group and compare them with patients who died by suicide who had not been to prison.

**Method:**

The clinical, sociodemographic and care characteristics of patients in contact with mental health services who died by suicide in the UK were examined in a national clinical survey between 1 January 2001 and 31 December 2021, and comparisons were made between former prisoners and patients with no history of being in prison.

**Results:**

Of the 33 381 (median age 46 years, range 10–100; 65.6% male) patients who died by suicide in the UK and had been in contact with mental health services in the 12 months before death, 3335 (11%) were ex-prisoners (male *n* = 2988, 90%; female *n* = 347, 10%). Compared with other patients, ex-prisoner patients had higher frequencies of personality disorder, schizophrenia and delusional disorders, as well as childhood abuse. Ex-prisoner patients were more likely to be male, to be aged between 45 and 65 years (median age 39, range 17–89), to live in deprived areas and to have a history of substance misuse. We found no differences in ethnicity.

**Conclusions:**

Mental health services need to focus particularly on patients with a history of being in prison who are experiencing economic adversity and offer substance-use-related interventions to ensure continued patient engagement. The link with deprivation is striking at a time at which rising costs of living are resulting in more health inequalities.

The UK prison population is approximately 90 000, with 159 prisoners per 100 000 of the population in England and Wales.^1^ Prisoners are a particularly vulnerable group in terms of suicide risk, as they have a higher likelihood of previous suicidality, psychiatric diagnoses, alcohol or drug use, and having experienced adverse life events including social isolation and financial difficulties.^2–4^ Former prisoners in England and Wales have been found to be at significantly greater risk of suicide than the general population, with the highest risk in the first 28 days after release.^5^ These findings are consistent across high-income countries.^6–8^ Among recently released prisoners, men are more likely to die by suicide than women, with other risk factors including White ethnicity, older age and a psychiatric diagnosis.^8^ There are several biological, environmental and societal risk factors associated with suicide during and after incarceration. Ex-prisoners have been identified as having higher rates of childhood abuse, developmental disorder, psychosis and affective disorders and use alcohol and drugs more compared with those who have never been to prison.^9^

Following release from prison, re-entry into the community, acquiring and maintaining employment, housing and reintegration with social networks can be significant challenges.^10^ Moreover, ex-prisoners more commonly live in low socioeconomic areas.^9^ As prisoners are maintained in a highly structured environment during their incarceration, their release into the community may be experienced as chaotic and destabilising; a personal need for structure has been reported to be a significant predictor of suicide attempts among ex-prisoners.^11^ Trauma, compounded by high rates of mental disorder, may contribute to the increased suicide risk within this population.^12^ There is a pressing need for integrative support for prisoners during their transition back into the community.^13^ This includes continuity of mental healthcare and transition of information across the criminal justice system and into community mental health services.^14,15^ However, although it has been found that distress typically persists in prisoners after release, contact with mental health services in the community seems to be minimal, particularly in the first 6 months after release.^16^ Drug and alcohol use among offender populations is well documented, with rates of problematic use being significantly higher in current and ex-prisoners compared with the general population.^17,18^ A history of drug and alcohol misuse is a well-known risk factor for suicide.^8,14^ Ex-prisoners have been identified as being particularly at risk for death by overdose after release from prison, often owing to decreased drug tolerance following a prolonged period of abstinence in prison.^19^

## Aims

It is important to examine which mental health services are used by ex-prisoners, as well as how they are used, to identify opportunities for intervention and inform strategies targeting the reduction of suicide in this population. In this study, we aimed to (a) explore former prisoners’ engagement with mental health services before their death by suicide; (b) examine the demographics, clinical history and clinical care of patients who died by suicide and were former prisoners; and (c) compare these characteristics with those of people who died by suicide who had not been to prison. We used suicide data from the National Confidential Inquiry into Suicide and Safety in Mental Health (NCISH) to examine differences in mental health patient characteristics between former prisoners and other patients who died by suicide.

## Method

This was an observational *post hoc* study analysing NCISH data on suicide by people in recent (past 12 months) contact with secondary mental health services. Data were collected for all people who died by suicide (including probable suicide) in England, Wales, Scotland and Northern Ireland between 1 January 2001 and 31 December 2021 while recently under the care of mental health services. A detailed description of NCISH data collection methods is available elsewhere.^20^ Briefly, data collection is undertaken in three stages. NCISH obtains information on all people who died by intentional self-harm or undetermined intent from general population mortality data from the Office for National Statistics. Mental health providers then identify which of these people had been in contact with mental health services in the 12 months before death (such cases are referred to as patient suicides). Detailed clinical information about each patient is then collected via a questionnaire completed by the clinician responsible for the patient’s care. Questionnaires collect information regarding the patient’s demographic characteristics, psychosocial history, details of suicide, treatment and adherence, last contact with services before death, and the clinician’s view on suicide prevention (additional details are provided in Supplementary Material 1 available at https://doi.org/10.1192/bjo.2025.71).

Data were compared between patients who had spent time in prison (hereafter referred to as ex-prisoner patients) and patients who had no prison history (hereafter referred to as patients), as recorded by the clinician completing the questionnaire. Patients who were in prison at the time of death (*n* = 198) were excluded from the analysis, as the focus of this study was ex-prisoners.

### Statistical analysis

Descriptive statistics are presented with frequencies and percentages. Percentages are expressed as valid percentages; i.e. if a response was missing, it was excluded from the analysis of that item. Owing to the time taken to register suicide deaths in the UK and the multiple stages of the NCISH methodology, temporal data in the most recent years (2019–2021) were projected to include cases that had been determined as likely to be patients and cases from unreturned questionnaires. Estimated numbers in the final year are presented as dotted lines in the figure. This is standard procedure in NCISH temporal data analyses, and the projected numbers generated through this method have been shown to be closely aligned with actual numbers, with a margin of error between 0 and 5%.^21^ The data used for the remaining analyses were the actual data collected rather than projected figures. To verify the results, we performed a sensitivity analysis.

Deprivation rank scores were calculated by linking postcodes of patients from the NCISH database with decile data within England, Scotland and Wales.^22–24^ To produce the deprivation rank scores, we used indices of multiple deprivation for each nation. Deprivation scores were excluded for Northern Ireland because the data were not available free of charge for our study. Each patient was assigned to a deprivation decile, with 1 being the most deprived and 10 the least deprived. For ease of interpretability, deciles were converted into quintiles. Odds ratios, both univariable and adjusted for age and sex, with 99% confidence intervals were calculated for all variables using logistic regression.

Variables with significant adjusted univariable logistic regressions were entered in a single step into a multivariable logistic regression model; the odds ratios were therefore adjusted for the presence of all other variables in the model. The dependent variable was coded as 0 (non-ex-prisoner) or 1 (ex-prisoner). Two multivariable logistic regression models were fitted: one that included forensic community team and probation service engagement at the time of death as predictors, and another that excluded these variables. This approach was taken because ex-prisoners were more likely to engage with these services, which could have biased the analysis of factors associated with the outcomes being studied. Owing to collinearity and a reduced range of data completeness (2011–2021), we omitted variables concerning specific drug types (as shown in Table 1). Similar to the approach used in descriptive analyses, we also omitted observations that contained a missing value for any of the model’s variables. Statistical significance was set at *P* ≤ 0.001 for both univariable and multivariable odds ratios, and Bonferroni correction (0.05/number of comparisons) was applied to correct for the large number of pairwise comparisons made. Data were analysed using Stata software, version 16 for Windows.

**Table 1:** Comparisons of drug and alcohol use by patients with no history of being in prison and ex-prisoner patients

Use of substances	Ex-prisoner patients *N* = 3335 % (*n*)	Patients *N* = 27 542% (*n*)	Unadjusted odds ratio^b^ (99% CI)	Adjusted by age and sex Odds ratio (99% CI)
Lifetime history of drug misuse	80 (2567)	29 (7656)	9.70 (8.62–10.90)*	7.79 (6.86–8.85)*
Lifetime history of alcohol misuse	77 (2489)	42 (11 268)	4.74 (4.23–5.31)*	3.59 (3.19–4.03)*
Drug misuse in <3 months	53 (1496)	16 (4028)	6.05 (5.43–6.73)*	4.57 (4.07–5.13)*
Alcohol misuse in <3 months	53 (1483)	27 (6881)	3.02 (2.72–3.35)*	2.35 (2.11–2.62)*
Contact with alcohol services^a^	19 (253)	7 (928)	2.90 (2.38–3.55)*	2.30 (1.87–2.84)*
Contact with drug services^a^	29 (394)	5 (671)	7.38 (6.14–8.89)*	5.59 (4.60–6.80)*
Evidence of increased substance use at last contact	28 (851)	12 (3228)	2.74 (2.44–3.07)*	2.13 (1.89–2.40)*
Patient discharged from mental health services owing to unresolved drug or alcohol use^a^	51 (150)	20 (535)	4.22 (3.05–5.84)*	3.18 (2.26–4.47)*
Lifetime misuse				
Heroin^a^	42 (523)	8 (741)	8.05 (6.73–9.62)*	6.76 (5.60–8.17)*
Stimulants^a^	43 (521)	17 (1505)	3.63 (3.07–4.28)*	2.89 (2.42–3.45)*
Benzodiazepines^a^	30 (341)	8 (729)	4.61 (3.9–5.59)*	4.18 (3.40–5.13)*
Cannabis^a^	48 (579)	22 (1935)	3.30 (2.81–3.89)*	2.72 (2.28–3.25)*
Skunk^a^	34 (102)	15 (255)	2.78 (1.94–3.98)*	2.65 (1.81–3.88)*
Legal high	23 (82)	9 (212)	3.02 (2.08–4.39)*	3.01 (2.01–4.50)*
Other^a^	9 (93)	4 (362)	2.23 (1.63–3.05)*	1.92 (1.39–2.66)*
Recent misuse				
Heroin	34 (364)	7 (538)	7.42 (6.06–9.08)*	5.91 (4.77–7.32)*
Stimulants	31 (324)	11 (890)	3.65 (3.00–4.44)*	2.79 (2.27–3.44)*
Benzodiazepines	25 (246)	7 (550)	4.45 (3.57–5.55)*	3.84 (3.04–4.86)*
Cannabis	38 (382)	16 (1282)	3.20 (2.66–3.85)*	2.52 (2.06–3.08)*
Skunk	21 (47)	11 (129)	2.21 (1.36–3.59)*	2.16 (1.28–3.64)
Legal high	12 (27)	8 (108)	1.42 (0.79–2.56)	1.35 (0.73–2.49)
Other	8 (69)	4 (283)	2.17 (1.51–3.10)*	1.82 (1.25–2.64)*

*P < 0.001.

a. Data available from 2011 to 2021 only for those with noted drug and/or alcohol misuse.

b. Calculated directly from the raw data without accounting for any confounders.

### Ethical approval

The authors assert that all procedures contributing to this work comply with the ethical standards of the relevant national and institutional committees on human experimentation and with the Helsinki Declaration of 1975, as revised in 2013. All procedures involving human participants and/or patients were approved by the National Research Ethics Service Committee Northwest (Greater Manchester South) (ERP 96/136). Exemption under section 251 of the NHS Act 2006, enabling access to confidential and identifiable information without consent in the interests of improving care, was obtained from the Health Research Agency Confidential Advisory Group.

## Results

**Fig. 1:**
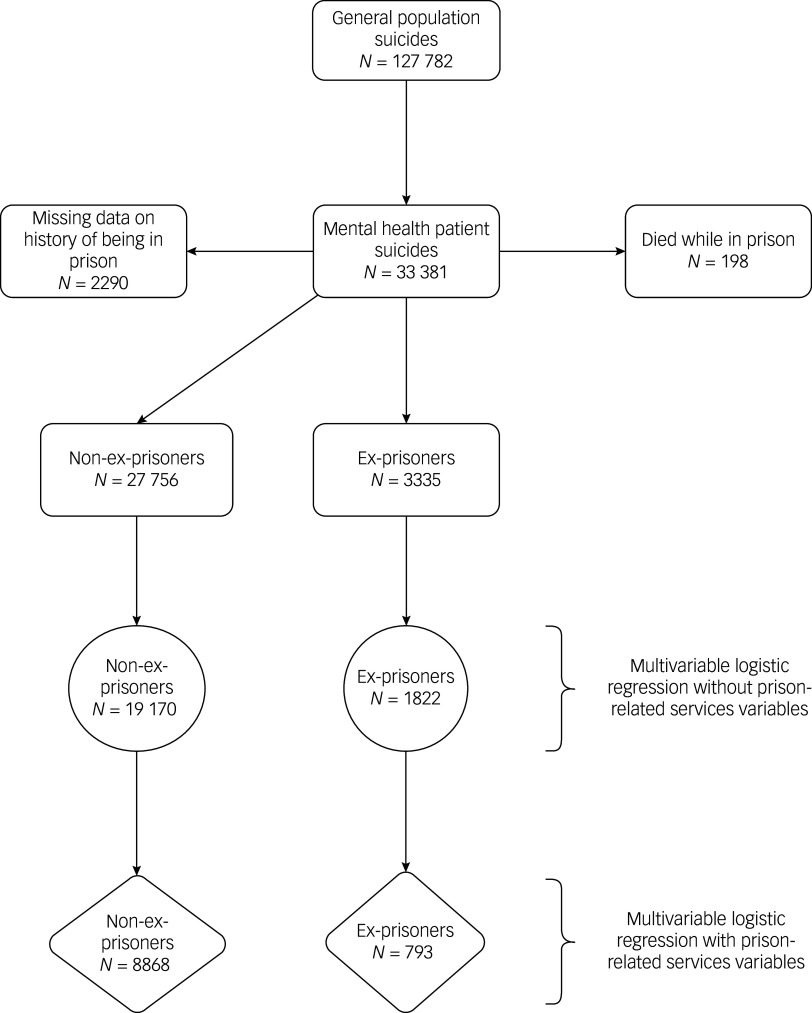
Data flow for the selection of sample of ex-prisoner patients.

Over the 21-year study period, NCISH were notified of 127 782 people who had died by suicide in England, Wales, Scotland, and Northern Ireland. Of those, 26% (*n* = 33 381) had been in contact with mental health services in the 12 months before their death. Eleven per cent (*n* = 3335) of patients were ex-prisoners (*n* = 3412 when projected), an average of 159 per year, and this proportion remained consistent over time (range: 8–13%) (Fig. 1). There were missing data regarding history of being in prison for 2290 (6.9%) patients who died by suicide during the study period (Fig. 1). Patients with missing data regarding history of being in prison were more likely to be male (76.1 *v*. 64.9%) and to have a diagnosis of alcohol (16.2 *v*. 8.2%) or drug (10.0 *v*. 4.8%) dependence. They were less likely to be between 25 and 44 years of age (40.0 *v*. 45.2%), to be in-patients at the time of death (4.2 *v*. 8.2%) and to die within 3 months post discharge (11.8 *v*. 17.8%). Sex-aggregated data are presented in Supplementary Material 2.

**Fig. 2:**
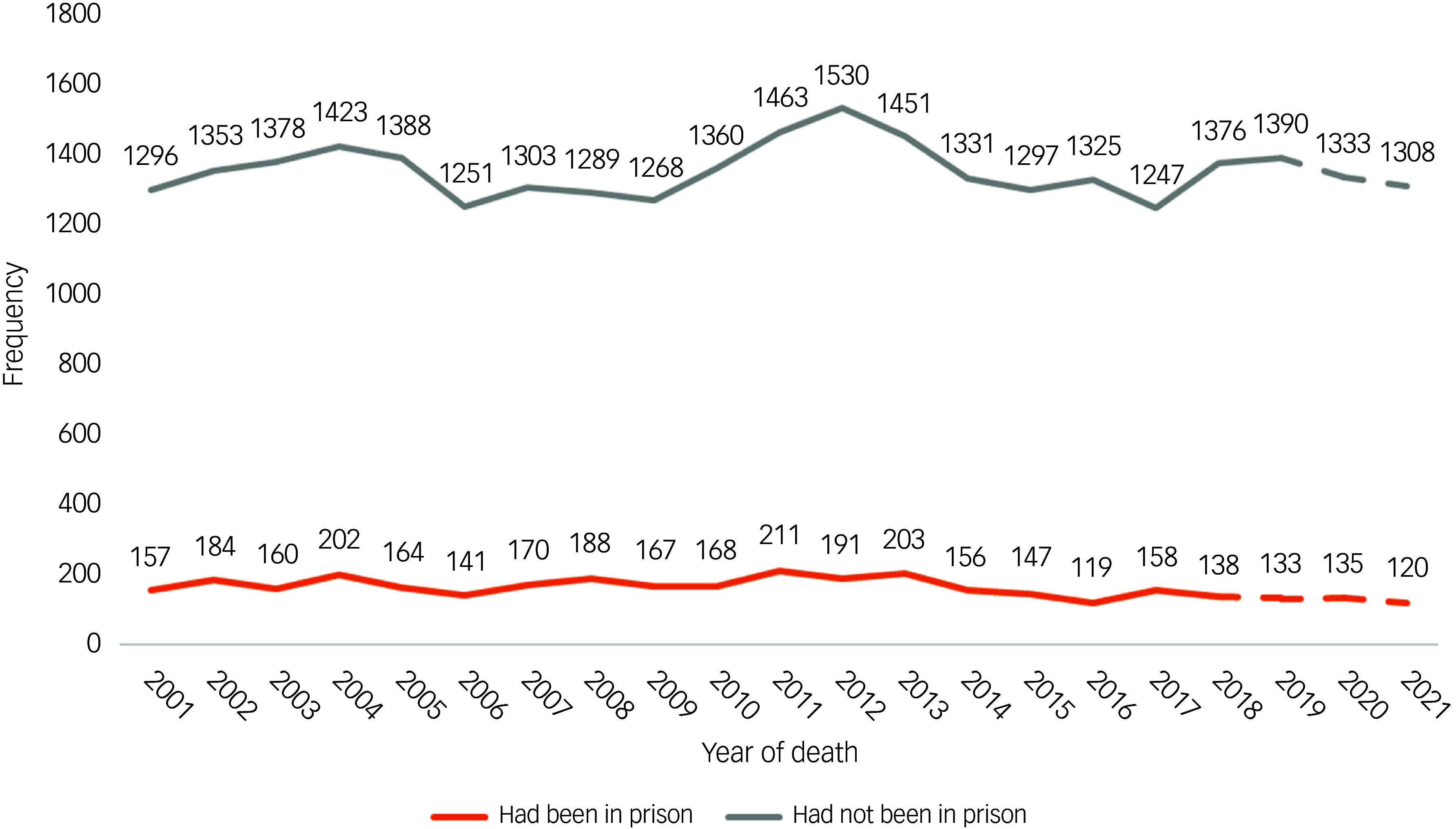
Proportions and numbers of patients who died by suicide who had or had not been reported to have spent time in prison before their death (2001–2021). Note that patient data were unavailable for Northern Ireland in 2020 and 2021.

Forty-three per cent (*n* = 1312) of ex-prisoner patients who died by suicide lived in the most deprived areas, compared with 25% (*n* = 6362) of patients who had no previous experience of being in prison. Five per cent (*n* = 157) of ex-prisoner patients who died by suicide lived in the least deprived areas (Fig. 2).

Compared with patients who had never been in prison, ex-prisoner patients were more likely to be aged between 25 and 44 years (odds ratio 2.65, 99% CI: 2.40–2.92), male (odds ratio 5.31, 95% CI: 4.57–6.17), unemployed (adjusted odds ratio 3.00, 99% CI: 2.68–3.36), unmarried (adjusted odds ratio 1.82, 95% CI: 1.59–2.08) and living alone (adjusted odds ratio 1.80, 99% CI: 1.62–2.00) (Table 2). In addition, ex-prisoner patients were more likely to be homeless (adjusted odds ratio 4.02, 95% CI: 3.23–5.01, *P* < 0.001) and to live in the most deprived areas (adjusted odds ratio 2.08, 99% CI: 1.88–2.32) compared with patients who were never prisoners.

**Table 2:** Comparisons between characteristics of patients and ex-prisoner patients: sociodemographic and suicide characteristics

Sociodemographic and suicide characteristics	Ex-prisoner patients *N* = 3335 % (*n*)	Patients *N* = 27 542 % (*n*)	Unadjusted odds ratio^a^ (99% CI)	Adjusted by age and sex Odds ratio (99% CI)
Age at death (years)				
Median (range)	39 (17–89)	46 (10–100)		–
Under 25	7 (233)	9 (2404)	Reference group	–
25–44	61 (2043)	37 (10 301)	2.05 (1.70–2.47)*	–
45–64	29 (981)	39 (10 819)	0.94 (0.77–1.14)*	–
65 and over	2 (69)	14 (3728)	0.20 (0.08–0.12)*	–
Ethnicity^b^				
Ethnic minority group	7 (214)	6 (1746)	1.02 (0.84–1.24)	0.89 (0.73–1.08)
Sex				
Male	90 (2988)	62 (17 037)	5.31 (4.57–6.17)*	–
Social factors				
Homeless	8 (259)	2 (422)	5.56 (4.51–6.85)*	4.02 (3.23–5.01)*
Living alone	61 (1883)	46 (12 117)	1.89 (1.71–2.09)*	1.80 (1.62–2.00)*.
Unmarried	84 (2638)	70 (18 782)	2.25 (1.97–2.56)*	1.82 (1.59–2.08)*
Unemployed	73 (2294)	41 (10 750)	3.96 (3.55–4.41)*	3.00 (2.68–3.36)*
Suicide method				
Hanging or strangulation	44 (1480)	43 (11 887)	(Reference group)	Reference group
Self-poisoning	30 (1001)	25 (6878)	1.17 (1.04–1.31)*	1.51 (1.34–1.70)*
Jumping from a height and/or in front of a vehicle	12 (387)	14 (3926)	0.79 (0.68–0.92)*	0.83 (0.70–0.97)
Drowning	4 (146)	6 (1644)	0.71 (0.56–0.95)*	1.05 (0.82–1.34)
Other	7 (235)	9 (2371)	0.80 (0.66–0.95)	0.84 (0.71–1.00)
Deprivation quintiles				
(Most deprived) 1st	43 (1312)	25 (6362)	5.47 (4.38–6.84)*	4.39 (3.50–5.51)*
2nd	27 (814)	22 (5708)	3.78 (3.00–4.77)*	3.25 (2.57–4.11)*
3rd	15 (450)	20 (5160)	2.31 (1.81–2.96)*	2.07 (1.61–2.65)*
4th	9 (284)	17 (4539)	1.66 (0.1.28–2.16)*	1.53 (1.17–2.00)*
(Least deprived) 5th	5 (157)	16 (4166)	(Reference group)	(Reference group)

*P < 0.0001.^1^

a. Calculated directly from the raw data without accounting for any confounders.

b. Ethnicity as collected available in Supplementary Material 3.

Ex-prisoner patients were more likely to die by self-poisoning (adjusted odds ratio 1.60, 99% CI: 1.43–1.78, *P* < 0.001) and less likely to die by jumping (adjusted odds ratio 0.74, 95% CI: 0.64–0.87, *P* < 0.001) compared with patients who had never been in prison. In terms of diagnoses, ex-prisoner patients were more likely to be diagnosed with drug dependence or misuse (adjusted odds ratio 5.49, 99% CI: 4.70–6.41), alcohol dependence or misuse (adjusted odds ratio 1.60, 99% CI: 1.38–1.86) or personality disorder (adjusted odds ratio 2.19, 95% CI: 1.90–2.54) compared with those that had not been to prison. Conversely, ex-prisoner patients were less likely to be diagnosed with affective disorders (adjusted odds ratio 0.29, 99% CI: 0.25–0.33) or other disorders (adjusted odds ratio 0.64, 95% CI: 0.55–0.75).

All types of childhood abuse (physical, emotional, sexual and unspecified) were more common in ex-prisoner patients (Table 3). As expected, before death, ex-prisoner patients were more commonly involved with probation (adjusted odds ratio 17.43, 99% CI: 12.30–24.70) and forensic (adjusted odds ratio 12.82, 95% CI: 6.70–24.51) services compared with patients who had not been to prison. Conversely, ex-prisoner patients were less likely to have accessed crisis resolution home treatment team (CRHT) services (adjusted odds ratio 0.48, 99% CI: 0.40–0.59), Improving Access to Psychological Treatment (IAPT) services (adjusted odds ratio 0.30, 95% CI: 0.15–0.58) or any other form of psychological treatment (adjusted odds ratio 0.60, 99% CI: 0.51–0.71).

**Table 3:** Comparisons between characteristics of patients and ex-prisoner patients: clinical characteristics

Clinical characteristics	Ex-prisoner patients *N* = 3335 % (*n*)	Patients *N* = 27 542 % (*n*)	Unadjusted odds ratio^b^ (99% CI)	Adjusted by age and sex Odds ratio (99% CI)
Primary psychiatric diagnosis				
Affective disorder	16 (538)	46 (12 448)	0.23 (0.20–0.26)*	0.29 (0.25–0.33)*
Schizophrenia and other delusional disorder	22 (713)	16 (4306)	1.46 (1.30–1.65)*	1.06 (0.94–1.20)
Personality disorder	16 (512)	9 (2490)	1.82 (1.59–2.08)*	2.19 (1.90–2.54)*
Alcohol dependence/misuse	14 (462)	8 (2048)	2.00 (1.73–2.30)*	1.60 (1.38–1.86)*
Drug dependenceor misuse	19 (627)	3 (821)	7.53 (6.50–8.71)*	5.49 (4.70–6.41)*
Other	11 (361)	16 (4370)	0.64 (0.55–0.75)*	0.64 (0.55–0.75)*
History of childhood abuse				
Physical^a^	21 (186)	8 (788)	2.97 (2.35–3.74)*	3.23 (2.52–4.13)*
Emotional^a^	26 (238)	14 (1424)	2.08 (1.69–2.57)*	2.43 (1.95–3.03)*
Sexual^a^	13 (114)	8 (742)	1.86 (1.41–2.46)*	2.77 (2.05–3.75)*
Unspecified^a^	29 (265)	14 (1348)	2.46 (2.01–3.02)*	2.86 (2.31–3.56)*
Clinical characteristics from				
In-patient mental health services	7 (221)	8 (2318)	0.77 (0.64–0.93)*	0.80 (0.66–0.96)
Crisis resolution home treatment team	6 (201)	13 (3407)	0.46 (0.38–0.56)*	0.48 (0.40–0.59)*
Subject to community treatment order at time of death	12 (33)	13 (166)	0.96 (0.57–1.62)	0.83 (0.48–1.44)
Early intervention services	2 (76)	3 (696)	0.92 (0.67–1.25)	0.66 (0.48–0.91)
Conveyed to a hospital-based place of safety under section 136 of the Mental Health Act	7 (65)	4 (359)	1.90 (1.32–2.72)*	1.64 (1.13–2.38)
Specialist personality disorder service^a^	2 (12)	1 (94)	1.28 (0.58–2.85)	2.02 (0.86–4.72)
Assertive outreach team	4 (130)	3 (680)	1.63 (1.27–2.10)*	1.30 (1.00–1.68)
Old people’s mental health service^a^	2 (24)	9 (1165)	0.17 (0.10–0.30)*	0.66 (0.32–1.33)
Forensic community teams^a^	3 (46)	<1 (31)	14.13 (7.73–25.83)*	12.82 (6.70–24.51)*
Probation service^a^	15 (190)	1 (100)	21.84 (15.72–30.33)*	17.43 (12.30–24.70)*
Improving Access to Psychological Therapies services^a^	1 (16)	4 (449)	0.32 (0.17–0.62)*	0.30 (0.15–0.58)*
Receiving any other psychological treatment	10 (294)	17 (4336)	0.53 (0.45–0.63)*	0.60 (0.51–0.71)*

*P < 0.0001.

a. Data available from 2011 to 2021.

b. Calculated directly from the raw data without accounting for any confounders.

Ex-prisoner patients were more likely to have had a history of drug and alcohol use, both historically (adjusted odds ratio 7.79, 99% CI: 6.86–8.85 and adjusted odds ratio 3.59, 95% CI: 3.19–4.03, respectively) and in the 3 months before death (adjusted odds ratio 4.57, 99% CI: 4.07–5.13 and adjusted odds ratio 2.35, 95% CI: 2.11–2.62). Furthermore, ex-prisoner patients were more likely to show evidence of increased use of substances at their last contact with services (adjusted odds ratio 2.13, 99% CI: 1.89–2.40) and to have been discharged from mental health services owing to unresolved problematic substance use (adjusted odds ratio 3.18, 95% CI: 2.26–4.47). Heroin, stimulants and cannabis were the most commonly used drugs in ex-prisoner patients, both historically and recently; with the exception of recent use of skunk and legal highs, ex-prisoner patients were more likely to have used all recorded substances, both historically and recently (Table 1).

A multivariable logistic regression analysis was conducted using statistically significant variables from the between-groups (patients with no history of being in prison and ex-prisoner patients) comparisons where data were available for the full study period (2001–2021). Final model findings are reported in Table 4. To examine the impact of predictors with and without contact with forensic or probation services, two models were fitted. On the basis of the results of the multivariable logistic regression, both with and without contact with forensic and probation services, those with a history of being in prison were more likely to be male, over 25 years of age, unemployed and homeless. They were also more likely to have a diagnosis of drug dependence and a lifetime history of both drug and alcohol misuse. In addition, they were more likely to live in the two most deprived quintiles (when using the least deprived quintile as a reference group). When contact with forensic and probation services was included in the analysis, these factors emerged as the strongest predictors, as expected. Conversely, when these predictors were excluded, patients with a history of being in prison were more likely to have a diagnosis of personality disorder and to belong to the third deprivation quintile.

**Table 4:** Main model findings of logistic regression for patients and ex-prisoner patients

Features and characteristics	Adjusted odds ratio^a^ (95% CI) (*N* = 9761)	Adjusted odds ratio^a^ (95% CI) Forensic community team and probation service excluded (*N* = 20 992)
Sociodemographic characteristics		
Sex		
Male	4.39 (3.40–5.67)*	4.44 (3.76–5.24)*
Age (years)		
Under 25	(Reference group)	(Reference group)
25–44	3.46 (2.24–5.33)*	2.61 (2.08–3.29)*
45–64	4.30 (2.75–6.72)*	2.72 (2.12–3.48)*
65 and over	3.90 (2.15–7.07)*	1.96 (1.35–2.86)*
Social factors		
Unemployed	1.76 (1.45–2.14)*	1.69 (1.50–1.90)*
Living alone	1.20 (0.97–1.47)	1.09 (0.96–1.24)
Unmarried	0.92 (0.70–1.20)	1.11 (0.93–1.31)
Homeless	2.70 (1.76–4.14)*	2.42 (1.86–3.15)*
Primary psychiatric diagnosis		
Affective disorder	0.57 (0.44–0.73)*	0.51 (0.43–0.60)*
Personality disorder	1.61 (1.20–2.14)	1.59 (1.32–1.91)*
Alcohol dependenceor misuse	1.09 (0.78–1.53)	1.03 (0.85–1.27)
Drug dependenceor misuse	2.53 (1.87–3.43)*	2.13 (1.75–2.60)*
Other diagnosis	0.91 (0.68–1.21)	0.85 (0.70–1.02)
Contact with services		
Crisis resolution home treatment team	0.68 (0.51–0.90)	0.65 (0.54–0.80)*
Forensic community teams	8.86 (4.38–17.92)*	–
Probation service	13.16 (9.02–19.20)*	–
Receiving any psychological treatment	0.90 (0.69–1.18)	0.87 (0.73–1.03)
Lifetime history of alcohol misuse	1.85 (1.44–2.39)*	1.94 (1.65–2.28)*
Lifetime history of drug misuse	4.40 (3.41–5.69)*	4.13 (3.51–4.84)*
Recent (<3 months) alcohol misuse	0.91 (0.71–1.16)	0.95 (0.81–1.11)
Recent (<3 months) drug misuse	0.89 (0.71–1.12)	0.98 (0.84–1.14)
Evidence of increased substance use at last contact	1.14 (0.91–1.41)	1.14 (0.99–1.30)
Deprivation quintiles		
(Most deprived) 1st	3.65 (2.48–5.36)*	2.77 (2.18–3.52)*
2nd	2.99 (2.02–4.43)*	2.45 (1.91–3.13)*
3rd	1.91 (1.26–2.91)	1.82 (1.41–2.36)*
4th	1.64 (1.05–2.55)	1.49 (1.13–1.97)
(Least deprived) 5th	(Reference group)	(Reference group)
Suicide methods		
Self-poisoning	1.18 (0.97–1.43)	1.22 (1.08–1.38)

a. Adjusted for the presence of all other variables in the model.

### Multivariable logistic regression by sex

Complete tables showing the results of the multivariable logistic regression split by sex are presented in Supplementary Material 2. When the multivariable regression models were fitted on male patients only, the outcomes mirrored those of the models that included all participants. Specifically, the direction and significance of the odds ratios for the predictors remained consistent between the male-only and full-participant models.

The results of the multivariable regression models fitted only on female patients, both with and without contact with forensic and probation services, consistently showed only diagnosis of drug dependence to be significantly predictive of a history of being in prison. As for the models including all and male-only patients, when contact with forensic and probation services was included in the analysis, these factors emerged as the strongest predictors. Finally, when these predictors were excluded, female patients with a history of being in prison were more likely to be between 25 and 44 years of age and to have a lifetime history of drug misuse.

## Discussion

### Summary of findings

This study aimed to examine the sociodemographic, clinical and care characteristics of ex-prisoner patients who had died by suicide. We found that one in nine people who died by suicide within 12 months of contact with mental health services had been in prison at some point in their lifetime. Patients who had a history of being in prison were more likely to have been living in highly deprived areas, were more often male, middle aged (aged 45 to 65 years), more likely to have experienced recent economic adversity and more likely to have a lifetime history of drug misuse compared with patients who died by suicide and had no history of being in prison.

This study also aimed to explore the engagement of ex-prisoner patients with mental health services before death by suicide over a 21-year period. We found that compared to other patients, ex-prisoner patients were more likely to have previously been diagnosed with personality disorder. However, there was no significant difference in engagement with specialist personality disorder services between ex-prisoner patients and patients who had no history of being in prison. Ex-prisoner patients were significantly less likely to have accessed mental health services that offer specialist support to suicidal patients, such as in-patient mental health services, CRHT services, or IAPT services. Similarly ex-prisoner patients were less likely to have received any psychological treatment. Ex-prisoner patients were more likely to be involved with forensic community teams and probation services, as would be expected for this population.

This study found that drug and alcohol use was significantly more prevalent in ex-prisoner patients compared with other patients. A lifetime history of drug and alcohol misuse and evidence of using substances at their last appointment with services were associated with suicide in ex-prisoner patients in the univariable analysis. Ex-prisoner patients were significantly more likely to have been involved with drug and alcohol services before their death, and half of ex-prisoner patients who had been discharged from mental health services before their death were discharged owing to problematic drug and/or alcohol use.

### Comparison with existing evidence and interpretation

More than one in nine patients who had been in contact with mental health services before their death had a history of being in prison at some point in their lifetime. This finding adds to existing research showing that former prisoners are a high-risk population for suicide.^5,7,12,25^ In examining sociodemographic risk factors for this population, we found that patients who had spent time in prison and died by suicide were more likely to be older than 25 and most likely to be men in mid-life, in line with previous research.^8^ Former prisoners are more likely to live in low socioeconomic areas; thus, re-entry into the community and seeking housing can be particularly difficult.^9,10^ We also found that former prisoner patients were more likely to be homeless at the time of death than patients who had no history of being in prison. Homeless patients experience more known risk factors for suicide compared with patients who have stable accommodation, including a history of drug and alcohol use.^26^ The current study found that more than two-thirds of patients with a history of being in prison who died by suicide had lived in the most deprived areas, with only 5% living in the least deprived areas. Unemployment is an established risk factor for suicide within the general population,^9,10,13^ and the current study also found unemployment to be a significant predictor for suicide among ex-prisoner patients. Other sociodemographic risk factors included being unmarried or living alone.

In line with previous findings, ex-prisoner patients were more likely to have been diagnosed with schizophrenia or delusional disorders and/or personality disorder and to have experienced childhood abuse compared with patients who did not have a history of being in prison.^9,12^ However, they were less likely to have experienced affective disorders, in contrast to findings of other studies.^9,27^ Previous research has shown that ex-prisoners tend to experience higher levels of distress but lower levels of engagement with clinical services compared with the general population, which has raised concerns about the continuity of care after release from prison.^16,28^ Likewise, the present study found that despite greater clinical need among ex-prisoner patients, access to most specialist services or psychological treatment was either the same as or less than that of the patient population overall. This included contact with CRHT services, which provide intensive support in the community to those experiencing crisis and have been shown to lower suicide rates.^29^ Despite alcohol and drug misuse being common among patients who die by suicide, only a minority are in contact with specialist alcohol and drug services before death.^21^ By contrast, we found that ex-prisoner patients were more likely than other patients to be in contact with specialist services; however, many who had been discharged from mental health services before death were discharged owing to drug and/or alcohol use. This points to an increased need for specialist services in this population and a need for joint working between mental health teams and community alcohol and drug teams to reduce suicide risk in this group.

### Methodological strengths and limitations

To our knowledge, this is the first national investigation of suicide in patients with a history of being in prison to have been conducted in the UK. The NCISH database is a large national suicide case series and contains robust representative clinical data, and the questionnaire response rate is high.^20^ However, the findings should be considered in the context of the following limitations. Our projected figures for 2019 and 2021 (the most incomplete years) may have been over- or underestimates. However, NCISH make similar projections every year for their annual report, and projected numbers are correlated closely with final numbers (margin of error: 0–5%).^30^ The NCISH data are subject to clinical judgement, which may be open to bias. This includes data on whether patients had been in prison before death, which were reported to us via the clinician completing the patient questionnaire rather than via the criminal justice service; therefore, forensic characteristics such as the nature of the offence and incarceration are not known. In addition, responses for some questions were unknown, resulting in missing data, which may have led to underestimation of some variables. Reporting of previous incarceration is likely to be an underestimate, as clinicians may be biased towards those who have been in prison more recently and those in whom it seems most relevant (patients given a diagnosis of personality disorder, those with a history of drug misuse, etc.) and may not be aware of historical incarceration; clinician bias may also lead to certain disorders (e.g. drug and alcohol use disorders and personality disorders) being diagnosed more frequently in ex-prisoners than in non-ex-prisoners. However, clinicians use both case notes and personal knowledge of the patient when completing questionnaires, and most of the questions are objective. NCISH data are a case series and do not infer causality. In addition, without a control group of ex-prisoners who died by suicide but had not been in recent contact with mental health services, we could not determine the relative importance of previous incarceration and contact with mental health services for suicide risk. Some drug-related deaths in ex-prisoner patients may have been due to returning to drug use after a period of being incarcerated, when tolerance of substances would have been reduced.^19^ However, in most cases, this will have been listed as an accidental death so is not likely to be recorded as a death from suicide on the NCISH database. In addition, the NCISH database has evolved in terms of the data that it collects over the past 25 years. Some data only began to be collected from 2011 onwards (e.g. the type of drug used) and some from 2016 onwards (information on whether the patient used legal highs), meaning that statistical tests could not capture all the data and that odds ratios may not accurately represent these variables. As such, the logistic regression model may have offered more predictors for ex-prisoner patient suicides had this information been available from 2001 to 2021. Information was not available on when the patient was last in prison, the number of episodes of imprisonment or the duration of imprisonment, and whether they were under probation at the time of death. This information would be useful for future research. Finally, the data presented here add to the wealth of research available in relation to former prisoner suicide in the UK and other high-income countries, although they may be less generalisable to lower-income countries.

### Implications

Previous research has found that it is particularly difficult for ex-prisoner patients to meet basic needs such as housing, support networks and employment, meaning that they continue to experience social adversity after release.^10,28^ There is a need for increased access to and engagement by services among patients who have been in prison to support their reintegration to the community and development of support networks, as this may reduce social isolation and increase their chances of obtaining and maintaining employment. Specialised services such as personality disorder treatment teams, CRHT, and early intervention and IAPT services should increase their accessibility to patients who have a history of being in prison and ensure that timely psychological treatment is provided to address clinical needs such as support with previous experience of trauma (childhood and imprisonment) and comorbid alcohol and/or drug misuse.

We found that some services were accessed more often by former prisoners; these included assertive outreach, services for those placed in a hospital-based place of safety under section 136 of the Mental Health Act as they posed a perceived threat to themselves or others owing to their mental disorder, and drug and alcohol services. Reviews of why these services are accessed by former prisoners and how they might better address the needs of ex-prisoners may improve engagement, retention and access. Ex-prisoners were discharged more often from services owing to drug and/or alcohol use, and there was evidence of substance misuse at appointments in the 3 months before their deaths. It would be useful to consider how services might engage with patients who experience distressing mental health problems alongside alcohol and drug use problems and, where third-sector alcohol and drug service providers are also involved in the patient’s care, how such teams can best collaborate with mental health services to ensure that alternatives to discharge can be found. The findings from this study support those of previous research that drug interventions and support are necessary for released prisoners. As former prisoners often do not engage in routine contact with health services, specialist teams should be established to provide support and treatment in addition to routine care to patients who are experiencing multiple adversities such as alcohol and/or drug use, homelessness, childhood trauma and suicidality, to encourage continued engagement. These teams should also help to ease the transition from prison to the general population for recently released ex-prisoners and their families, as well as helping them to get in touch with charities and local organisations that can support their wider health and well-being needs in addition to their mental health needs, for instance, by providing support with housing, relationships, finances, employment, and drug and alcohol misuse and social support. This is particularly important given the finding that more than two-thirds of ex-prisoner patients were living in the most deprived areas at the time of death (first and second quintiles).

## Supporting information

Baird et al. supplementary material 1 Baird et al. supplementary material

Baird et al. supplementary material 2 Baird et al. supplementary material

Baird et al. supplementary material 3 Baird et al. supplementary material

## Data Availability

Electronic health records are, by definition, considered sensitive data in the UK by the Data Protection Act and cannot be shared via public deposition because of information governance restrictions in place to protect patient confidentiality. Access to Office for National Statistics data is subject to request. For more information, see https://www.ons.gov.uk/aboutus/whatwedo/statistics/requestingstatistics.

## Supplementary material

The supplementary material is available online at https://doi.org/10.1192/bjo.2025.71
