# Supplementary material for: An examination of former prisoners’ mental health problems before death by suicide over a 21-year period (2001–2021)
Source: BJPsych Open. 2025 Jun 23;11(4):e124. doi: 10.1192/bjo.2025.71 (PMC12188233; doi:10.1192/bjo.2025.71)
Supplement: Baird et al. supplementary material 1 — Baird et al. supplementary material [file S2056472425000717sup001.docx]

**Appendix 1: Detailed ethnicity**

| Non-white ethnicity | **Ex-prisoner patients**  **N=214**  **% (n)** | **Patients**  **N=1,746**  **% (n)** | **Unadjusted OR (99% CI)** |
| --- | --- | --- | --- |
| Black African | 14 (30) | 12 (215) | 1.16 (0.68-1.99) |
| Black Caribbean | 20 (43) | 9 (155) | 2.58 (1.58-4.21)* |
| Black British | 3 (7) | 3 (54) | 1.06 (0.37-3.03) |
| Indian/Pakistani/Bangladeshi | 8 (17) | 12 (217) | 0.61 (0.31-1.20) |
| Asian British | 12 (26) | 22 (391) | 0.48 (0.27-0.84) |
| Chinese | < 3 | 4 (71) | 0.34 (0.07-1.55) |
| Mixed/multiple ethnicity | 26 (56) | 20 (355) | 1.39 (0.90-2.13) |
| Arab/Middle Eastern | 7 (15) | 5 (86) | 1.45 (0.69-3.07) |
| Other | 8 (17) | 12 (202) | 0.66 (0.33-1.30) |

*p<0.001
