# Supplementary material for: An examination of former prisoners’ mental health problems before death by suicide over a 21-year period (2001–2021)
Source: BJPsych Open. 2025 Jun 23;11(4):e124. doi: 10.1192/bjo.2025.71 (PMC12188233; doi:10.1192/bjo.2025.71)
Supplement: Baird et al. supplementary material 2 — Baird et al. supplementary material [file S2056472425000717sup002.docx]

**Appendix 2. Analysis by sex: Male**

**Table 1. Comparisons between characteristics of patients and ex-prisoner patients-sociodemographic and suicide characteristics**

|  | Ex-prisoner patients  N=2,988  % (n) | Patients  N=17,037  % (n) | Unadjusted OR^†^ (99% CI) | Adjusted by age and sex  OR (99% CI) |
| --- | --- | --- | --- | --- |
| Age at death (years) |  |  |  |  |
| Median(range) | 39 (17-89) | 45 (10-100) |  | - |
| Under 25 | 7 (206) | 9 (1,483) | 0.78 (0.64-0.95) | - |
| 25 – 44 | 61 (1,816) | 39 (6,645) | 2·42 (2·18-2·69)* | - |
| 45-64 | 30 (894) | 38 (6,557) | 0·68 (0·61-0·76)* | - |
| 65 and over | 2 (63) | 13 (2,196) | 0·15 (0·10-0·20)* | - |
| Ethnicity |  |  |  |  |
| Ethnic minority group^ | 7 (190) | 7 (1,087) | 1·00 (0·81-1·24) | 0.88 (0.71-1.09) |
| White | 93 (2,715) | 93 (15,567) | 1.00 (0.81-1.23) | 1.13 (0.91-1.40) |
| Social factors |  |  |  |  |
| Homeless | 9 (245) | 2 (323) | 4·74 (3·78-5·93)* | 4.07 (3.24-5.12)* |
| Living alone | 62 (1,697) | 47 (7,772) | 1·79 (1·61-1.99)* | 1.76 (1.58-1.98)*. |
| Unmarried | 84 (2,359) | 72 (11,895) | 2·09 (1·82-2·41)* | 1.76 (1.52-2.03)* |
| Unemployed | 73 (2,056) | 43 (6,947) | 3·70 (3·29-4·16)* | 2.97 (2.63-3.36)* |
|  |  |  |  |  |
| Suicide method |  |  |  |  |
| Hanging/Strangulation | 46 (1,370) | 48 (8,185) | 0·92 (0·83-1·01) | 0.91 (0.82-1.01) |
| Self-poisoning | 28 (847) | 19 (3,239) | 1·69 (1·50-1·89)* | 1.61 (1.43-1.81)* |
| Jumping from a height/in front of a vehicle | 12 (347) | 15 (2,504) | 0·76 (0·65-0·89)* | 0.74 (0.63-0.86)* |
| Drowning | 5 (135) | 5 (894) | 0·85 (0·67-1·10) | 1.03 (0.81-1.33) |
| Other | 7 (209) | 9 (1,569) | 0·74 (0·61-0·90)* | 0.82 (0.67-1.00) |
| Deprivation quintiles |  |  |  |  |
| (most deprived) 1^st^ | 43 (1,171) | 25 (4,954) | 2·26 (2·02-2·52)* | 2.06 (1.84-2.30)* |
| 2^nd^ | 27 (720) | 22 (3,479) | 1·31 (1·16-1·48)* | 1.25 (1.11-1.43)* |
| 3^rd^ | 15 (415) | 20 (3,204) | 0·73 (0·63-0·84)* | 0.75 (0.65-0.87)* |
| 4^th^ | 9 (255) | 18 (2,821) | 0·49 (0·41-0·58)* | 0.52 (0.43-0.62)* |
| (least deprived) 5^th^ | 5 (137) | 15 (2,436) | 0·30 (0·24-0·38)* | 0.33 (0.26-0.42)* |

^*^p<0.0001

^Ethnicity as collected available in Appendix 1

^†^calculated directly from the raw data without accounting for any confounders

**Table 2. Comparisons between characteristics of patients and ex-prisoner patient-clinical characteristics**

|  | Ex-prisoner patients  N=2,988  % (n) | Patients  N=17,037  % (n) | Unadjusted OR^†^ (99% CI) | Adjusted by age and sex  OR (99% CI) |
| --- | --- | --- | --- | --- |
| Primary psychiatric diagnosis |  |  |  |  |
| Affective disorder | 16 (483) | 44 (7,303) | 0·25 (0·22-0·29)* | 0.30 (0.26-0.34)* |
| Schizophrenia and other delusional disorder | 22 (663) | 19 (3,103) | 1·28 (1·13-1·44)* | 1.06 (0.94-1.21) |
| Personality disorder | 14 (402) | 6 (974) | 2·55 (2·17-3·01)* | 2.21 (1.87-2.60)* |
| Alcohol dependence/misuse | 14 (426) | 9 (1,455) | 1.77 (1·52-2·07)* | 1.60 (1.37-1.87)* |
| Drug dependence/misuse | 19 (556) | 4 (592) | 6·34 (5·39-7·46)* | 5.11 (4.33-6.03)* |
| Other | 12 (340) | 17 (2,847) | 0·64 (0·54-0·75)* | 0.67 (0.57-0.79)* |
| History of childhood abuse |  |  |  |  |
| Physical^1^ | 20 (163) | 7 (421) | 3.43 (2·64-4·45)* | 3.21 (2.46-4.18)* |
| Emotional^1^ | 24 (201) | 11 (712) | 2·50 (1·98-3·15)* | 2.32 (1.83-2.94)* |
| Sexual^1^ | 11 (86) | 4 (256) | 2·88 (2·05-4·03)* | 2.56 (1.82-3.60)* |
| Unspecified^1^ | 26 (218) | 10 (633) | 3·06 (2.43-3·85)* | 2.70 (2.13-3.41)* |
| Clinical characteristics from questionnaire data |  |  |  |  |
| In-patient mental health services | 7 (204) | 8 (1,339) | 0·86 (0·70-1·05) | 0.84 (0.69-1.03) |
| Crisis Resolution Home Treatment Team (CRHT) | 6 (177) | 13 (2,084) | 0·46 (0·37-0·57)* | 0.47 (0.38-0.58)* |
| Subject to Community Treatment Order (CTO) at time of death | 12 (29) | 14 (103) | 0·87 (0·49-1·55) | 0.78 (0.43-1.40) |
| Early Intervention (EI) services | 3 (71) | 3 (524) | 0·78 (0·56-1·09) | 0.67 (0.48-0.93) |
| Conveyed to a hospital-based place of safety under S136 of MHA | 7 (55) | 4 (229) | 1·72 (1·15-2·56)* | 1.50 (1.00-2.25) |
| Specialist Personality Disorder Service ^1^ | 2 (10) | 1 (22) | 3·15 (1·17-8.47) | 2.93 (1.06-8.15) |
| Assertive Outreach team | 4 (118) | 3 (444) | 1·57 (1·19-2·07)* | 1.29 (0.98-1.71) |
| Old people’s mental health service^1^ | 2 (20) | 9 (729) | 0·16 (0·09-0·29)* | 0.55 (0.26-1.18) |
| Forensic community teams^1^ | 3 (42) | <1 (25) | 11·12 (5·77-21·41)* | 11.71 (5.92-23.17)* |
| Probation service^1^ | 15 (172) | 1 (84) | 16·37 (11·49-23·32)* | 15.62 (10.82-22.54)* |
| IAPT services^1^ | 1 (15) | 4 (292) | 0·32 (0·17-0·64)* | 0.31 (0.16-0.62)* |
| Receiving any other psychological treatment | 9 (247) | 15 (2,343) | 0·58 (0·48-0·70)* | 0.59 (0.49-0.71)* |

*^1^*Data available from 2011-2021

^†^calculated directly from the raw data without accounting for any confounders

**Table 3. Comparisons between drug and alcohol use by patients with no history of being in prison and ex-prisoner patients**

|  | Ex-prisoner patients  N=2,988  % (n) | Patients  N=17,037  % (n) | Unadjusted OR^†^ (99% CI) | Adjusted by age and sex  OR (99% CI) |
| --- | --- | --- | --- | --- |
| Lifetime history of drug misuse | 80 (2,313) | 32 (5,387) | 8·31 (7·32-9·43)* | 7.91 (6.72-8.84)* |
| Lifetime history of alcohol misuse | 77 (2,231) | 46 (7,601) | 4·06 (3·60-4·59)* | 3.43 (3.03-3.88)* |
| Drug misuse in <3mths | 53 (1,342) | 18 (2,872) | 5·09 (4·53-5·71)* | 4.36 (3.85-4.93)* |
| Alcohol misuse in <3mths | 53 (1,321) | 30 (4,658) | 2·63 (2·35-2·95)* | 2.25 (2.01-2.52)* |
| Contact with alcohol service^1^ | 18 (219) | 8 (621) | 2·58 (2·07-3·22)* | 2.17 (1.73-2.71)* |
| Contact with drug service^1^ | 29 (346) | 6 (472) | 6·33 (5·16-7.76)* | 5.21 (4.23-6.43)* |
| Evidence of increased substance use at last contact | 28 (766) | 14 (2,237) | 2·42 (2·13-2·73)* | 2.07 (1.82-2.35)* |
| Pt discharged from MH services to unresolved drug/alcohol use^1^ | 51 (131) | 24 (386) | 3·29 (2·31-4.69)* | 2.88 (2.00-4.15)* |
| Lifetime misuse |  |  |  |  |
| Heroin^1^ | 42 (462) | 9 (486) | 7·62 (6·25-9·30)* | 6.52 (5.33-7.99)* |
| Stimulants^1^ | 42 (463) | 19 (1,101) | 3·05 (2·54-3·65)* | 2.73 (2.25-3.29)* |
| Benzodiazepines^1^ | 29 (301) | 8 (448) | 4·70 (3·78-5·84)* | 4.17 (3.34-5.21)* |
| Cannabis^1^ | 48 (521) | 25 (1,426) | 2·75 (2·31-3·28)* | 2.60 (2.16-3.14)* |
| Skunk^1^ | 32 (88) | 18 (201) | 2.10 (1.42-3·09)* | 2.26 (1.51-3.38)* |
| Legal high | 23 (76) | 11 (166) | 2·56 (1·72-3·81)* | 2.95 (1.93-4.51)* |
| Other^1^ | 9 (81) | 5 (245) | 2·02 (1·43-2·85)* | 1.82 (1.30-2.58)* |
| Recent misuse |  |  |  |  |
| Heroin | 34 (320) | 7 (365) | 6·75 (5·38-8·45)* | 5.64 (4.48-7.11)* |
| Stimulants | 31 (286) | 13 (663) | 2·98 (2·41-3·69)* | 2.58 (2.08-3.23)* |
| Benzodiazepines | 24 (217) | 7 (344) | 4·45 (3·47-5.69)* | 3.83 (2.97-4.94)* |
| Cannabis | 38 (345) | 19 (966) | 2·62 (2·15-3·19)* | 2.41 (1.95-2.98)* |
| Skunk | 21 (42) | 13 (101) | 1·75 (1·03-2·96) | 1.98 (1.14-3.43) |
| Legal high | 13 (27) | 10 (82) | 1·37 (0.74-2·52) | 1.52 (0.81-2.85) |
| Other | 7 (61) | 4 (194) | 1·96 (1·33-2·90)* | 1.76 (1.18-2.62)* |

*p<0.01 *^1^*Data available from 2011-2021 only for those with noted drugs/alcohol misuse

^†^calculated directly from the raw data without accounting for any confounders

**Table 4. Main model findings of logistic regression for patients and ex-prisoner patients**

|  | Adjusted OR  (95% CI)  (N=6,239) | Adjusted OR  (95% CI)  Forensic community team and probation service excluded  (N=13,348) |
| --- | --- | --- |
| Constant | 0·003 (0·001-0·006) | 0·01 (0·004-0·01) |
| Sociodemographic characteristics |  |  |
| Age |  |  |
| Under 25 | (reference group) | (reference group) |
| 25-44 | 3·47 (2·18-5·54)* | 2.56 (2.00-3.29)* |
| 45-64 | 4.57 (2·83-7·39)* | 2.78 (2.13-3.63)* |
| 65 and over | 3·96 (2·08-7·51)* | 2.05 (1.38-3.07)* |
| Unemployed | 1·75 (1·42-2·15)* | 1.71 (1.50-1.94)* |
| Living alone | 1.18 (0.95-1.47) | 1.09 (0.95-1.25) |
| Unmarried | 0.99 (0.74-1.32) | 1.08 (0.90-1.29) |
| Homeless | 2.87 (1.84-4.49)* | 2.66 (2.02-3.52)* |
| Primary psychiatric diagnosis |  |  |
| Affective disorder | 0.59 (0.45-0.78)* | 0.54 (0.45-0.64)* |
| Personality disorder | 1.60 (1.16-2.22) | 1.60 (1.30-1.97)* |
| Alcohol dependence/misuse | 1.06 (0.74-1.51) | 1.04 (0.84-1.29) |
| Drug dependence/misuse | 2·22 (1·60-3·07)* | 1.90 (1.54-2.34)* |
| Other diagnosis | 0.94 (0.70-1.27) | 0.88 (0.72-1.08) |
| Contact with services |  |  |
| Crisis Resolution Home Treatment Team (CRHT) | 0.68 (0.50-0.92) | 0.64 (0.52-0.79)* |
| Forensic community teams | 7.66 (3·71-15·82)* | - |
| Probation service | 11·80 (7·84-17·75)* | - |
| Receiving any psychological treatment | 0.89 (0.66-1.20) | 0.88 (0.73-1.06) |
| Lifetime history of alcohol misuse | 1.79 (1.37-2.35)* | 4.36 (3.68-5.18)* |
| Lifetime history of drug misuse | 4.84 (3·68-6·37)* | 1.91 (1.61-2.27)* |
| Recent (<3 months) alcohol misuse | 0.95 (0.73-1.23) | 0.92 (0.78-1.09) |
| Recent (<3 months) drug misuse | 0.86 (0.67-1.10) | 0.97 (0.97-1.30) |
| Evidence of increased substance use at last contact | 1.05 (0.83-1.32) | 1.13 (0.97-1.30) |
| Deprivation quintiles |  |  |
| 1 | 3.44 (2.31-5.14)* | 2.75 (2.13-3.55)* |
| 2 | 2.89 (1.92-4.35)* | 2.43 (1.87-3.15)* |
| 3 | 1.90 (1.23-2.94) | 1.91 (1.45-2.50)* |
| 4 | 1.57 (0.99-2.49) | 1.50 (1.12-2.00) |
| 5 | (reference group) | (reference group) |
| Suicide methods |  |  |
| Self-poisoning | 1.09 (0.87-1.40) | 1.18 (1.03-1.35) |
| Jumping | 0.79 (0.59-1.06) | 0.89 (0.75-1.06) |

**By sex: Female**

**Table 1. Comparisons between characteristics of patients and ex-prisoner patients-sociodemographic and suicide characteristics**

|  | Ex-prisoner patients  N=347  % (n) | Patients  N=10,505  % (n) | Unadjusted OR^†^ (99% CI) | Adjusted by age  OR (99% CI) |
| --- | --- | --- | --- | --- |
| Age at death (years) |  |  |  |  |
| Median(range) | 37 (17-85) | 47 (12-97) |  | - |
| Under 25 | 8 (27) | 9 (921) | 0.88 (0.52-1.48) | - |
| 25 – 44 | 65 (227) | 35 (3,656) | 3·54 (2·64-4·76)* | - |
| 45-64 | 25 (87) | 41 (4,262) | 0·49 (0·35-0·68)* | - |
| 65 and over | 2 (6) | 15 (1,532) | 0·10 (0·04-0·30)* | - |
| Ethnicity |  |  |  |  |
| Ethnic minority group^ | 7 (24) | 6 (659) | 1·11 (0·64-1·94) | 0.93 (0.53-1.63) |
| White | 93 (314) | 94 (9,598) | 0.90 (0.52-1.57) | 1.08 (0.61-1.88) |
| Social factors |  |  |  |  |
| Homeless | 4 (14) | 1 (99) | 4·53 (2·14-9·58)* | 3.52 (1.64-7.53)* |
| Living alone | 58 (186) | 43 (4,345) | 1·84 (1·37-2.47)* | 2.08 (1.54-2.81)*. |
| Unmarried | 84 (279) | 68 (6,887) | 2·47 (1·68-3·63)* | 2.30 (1.55-3.40)* |
| Unemployed | 73 (238) | 38 (3,803) | 4·32 (3·12-5·96)* | 3.19 (2.29-4.45)* |
|  |  |  |  |  |
| Suicide method |  |  |  |  |
| Hanging/Strangulation | 32 (110) | 35 (3,702) | 0·86 (0·63-1·16) | 0.73 (0.54-0.99) |
| Self-poisoning | 45 (154) | 35 (3,639) | 1·51 (1·14-2·01)* | 1.54 (1.16-2.05)* |
| Jumping from a height/in front of a vehicle | 12 (40) | 14 (1,422) | 0·83 (0·54-1·30) | 0.84 (0.54-1.30) |
| Drowning | 3 (11) | 7 (750) | 0·43 (0·19-0·95) | 0.67 (0.30-1.51) |
| Other | 8 (26) | 8 (802) | 0·98 (0·58-1·68) | 1.17 (0.68-2.01) |
| Deprivation quintiles |  |  |  |  |
| (most deprived) 1^st^ | 44 (141) | 23 (2,308) | 2·62 (1·95-3·52)* | 2.25 (1.67-3.04)* |
| 2^nd^ | 29 (94) | 22 (2,229) | 1·45 (1·04-2·00) | 1.32 (0.96-1.83) |
| 3^rd^ | 11 (35) | 20 (1,956) | 0·50 (0·32-0·80)* | 0.53 (0.33-0.84)* |
| 4^th^ | 9 (29) | 17 (1,718) | 0·48 (0·29-0·79)* | 0.53 (0.32-0.88)* |
| (least deprived) 5^th^ | 6 (20) | 17 (1,730) | 0·32 (0·17-0·58)* | 0.38 (0.21-0.69)* |

^*^p<0.0001

^Ethnicity as collected available in Appendix 1

^†^calculated directly from the raw data without accounting for any confounders

**Table 2. Comparisons between characteristics of patients and ex-prisoner patient-clinical characteristics**

|  | Ex-prisoner patients N=347  % (n) | Patients  N=10,505  % (n) | Unadjusted OR^†^ (99% CI) | Adjusted by age  OR (99% CI) |
| --- | --- | --- | --- | --- |
| Primary psychiatric diagnosis |  |  |  |  |
| Affective disorder | 16 (55) | 50 (5,145) | 0·19 (0·13-0·28)* | 0.23 (0.16-0.35)* |
| Schizophrenia and other delusional disorder | 14 (50) | 12 (1,203) | 1·29 (0·86-1·93) | 1.20 (0.80-1.80) |
| Personality disorder | 32 (110) | 15 (1,516) | 2·73 (2·01-3·70)* | 2.06 (1.50-2.81)* |
| Alcohol dependence/misuse | 10 (36) | 6 (593) | 1.92 (1·20-3·06)* | 1.58 (0.99-2.54) |
| Drug dependence/misuse | 21 (71) | 2 (229) | 11·46 (7·81-16·81)* | 8.31 (5.61-12.32)* |
| Other | 6 (21) | 15 (1,523) | 0·38 (0·21-0·68)* | 0.41 (0.23-0.74)* |
| History of childhood abuse |  |  |  |  |
| Physical^1^ | 31 (23) | 10 (367) | 3.77 (1·95-7·30)* | 3.21 (1.64-6.26)* |
| Emotional^1^ | 47 (37) | 20 (712) | 3·48 (1·93-6·29)* | 3.18 (1.74-5.80)* |
| Sexual^1^ | 41 (28) | 14 (486) | 4·22 (2·22-8·04)* | 3.65 (1.90-7.03)* |
| Unspecified^1^ | 58 (47) | 21 (715) | 5·16 (2.86-9·31)* | 4.05 (2.22-7.39)* |
| Clinical characteristics from questionnaire data |  |  |  |  |
| In-patient mental health services | 5 (17) | 9 (979) | 0·50 (0·26-0.96) | 0.50 (0.26-0.95) |
| Crisis Resolution Home Treatment Team (CRHT) | 7 (24) | 13 (1,323) | 0·52 (0·30-0·90) | 0.55 (0.32-0.96) |
| Subject to Community Treatment Order (CTO) at time of death | 15 (4) | 12 (63) | 1·32 (0·31-5·57) | 1.40 (0.33-6.02) |
| Early Intervention (EI) services | 2 (5) | 2 (172) | 0·87 (0·27-2·83) | 0.63 (0.19-2.06) |
| Conveyed to a hospital-based place of safety under S136 of MHA | 11 (10) | 4 (130) | 3·24 (1·33-7·93)* | 2.78 (1.12-6.87) |
| Specialist Personality Disorder Service ^1^ | 3 (<3) | 3 (72) | 1·03 (0·16-6.72) | 0.82 (0.12-5.39) |
| Assertive Outreach team | 4 (12) | 2 (236) | 1·56 (0·72-3·39) | 1.38 (0.63-3.02) |
| Old people’s mental health service^1^ | 3 (4) | 9 (436) | 0·28 (0·08-1·05) | 4.01 (0.34-46.86) |
| Forensic community teams^1^ | 3 (4) | <1 (6) | 22·61 (4·23-120·93)* | 23.16 (3.99-134.58)* |
| Probation service^1^ | 153 (18) | <1 (16) | 44·58 (17·83-111·47)* | 35.20 (13.61-91.05)* |
| IAPT services^1^ | 1 (<3) | 3 (157) | 0·20 (0·15-2.73) | 0.17 (0.01-2.35) |
| Receiving any psychological treatment | 15 (47) | 20 (1,916) | 0·69 (0·46-1·04) | 0.63 (0.42-0.96) |

*^1^*Data available from 2011-2021

^†^calculated directly from the raw data without accounting for any confounders

**Table 3. Comparisons between drug and alcohol use by patients with no history of being in prison and ex-prisoner patients**

|  | Ex-prisoner patients N=347% (n) | Patients  N=10,505  % (n) | Unadjusted OR^†^ (99% CI) | Adjusted by age  OR (99% CI) |
| --- | --- | --- | --- | --- |
| Lifetime history of drug misuse | 75 (254) | 22 (2,269) | 10·71 (7·69-14·92)* | 8.39 (5.93-11.88)* |
| Lifetime history of alcohol misuse | 78 (258) | 36 (3,667) | 6·22 (4·41-8·77)* | 4.84 (3.41-6.86)* |
| Drug misuse in <3mths | 52 (154) | 12 (1,156) | 8·20 (6·01-11·19)* | 6.40 (4.62-8.87)* |
| Alcohol misuse in <3mths | 55 (162) | 23 (2,223) | 4·06 (2·98-5·51)* | 3.18 (2.33-4.34)* |
| Contact with alcohol service^1^ | 18 (219) | 8 (621) | 2·58 (2·07-3·22)* | 2.17 (1.73-2.71)* |
| Contact with drug service^1^ | 23 (34) | 6 (307) | 4·42 (2·61-7.49)* | 3.33 (1.94-5.69)* |
| Evidence of increased substance use at last contact | 27 (85) | 10 (991) | 3·34 (2·38-4·69)* | 2.63 (1.87-3.71)* |
| Pt discharged from MH services to unresolved drug/alcohol use^1^ | 51 (19) | 14 (149) | 6·68 (2·78-16.06)* | 5.82 (2.37-14.28)* |
| Lifetime misuse |  |  |  |  |
| Heroin^1^ | 48 (61) | 8 (255) | 10·60 (6·51-17·26)* | 7.94 (4.80-13.12)* |
| Stimulants^1^ | 45 (58) | 13 (404) | 5·61 (3·49-9·03)* | 4.26 (2.57-7.06)* |
| Benzodiazepines^1^ | 34 (40) | 9 (281) | 5·15 (3·04-8·70)* | 4.14 (2.42-7.07)* |
| Cannabis^1^ | 48 (58) | 16 (509) | 4·73 (2·92-7·67)* | 3.79 (2.28-6.30)* |
| Skunk^1^ | 50 (14) | 10 (54) | 9.28 (3.28-26·28)* | 9.39 (3.11-28.30)* |
| Legal high | 20 (6) | 6 (46) | 4.07 (1·18-14·05) | 4.03 (1.12-14.46) |
| Other^1^ | 12 (12) | 4 (117) | 3·26 (1·43-7·46)* | 2.81 (1.21-6.51) |
| Recent misuse |  |  |  |  |
| Heroin | 39 (44) | 6 (173) | 10·20 (5·97-17·42)* | 7.34 (4.21-12.78)* |
| Stimulants | 38 (34) | 8 (227) | 6.29 (3·65-10·85)* | 4.88 (2.74-8.70)* |
| Benzodiazepines | 29 (27) | 7 (206) | 4·88 (2·71-8.80)* | 3.86 (2.11-7.04)* |
| Cannabis | 36 (37) | 11 (316) | 4·62 (2·67-8·01)* | 3.73 (2.10-6.63)* |
| Skunk | 24 (5) | 7 (28) | 4·33 (1·05-17·78) | 4.77 (1.09-20.83) |
| Legal high | 0 | 6 (26) | - | - |
| Other | 8 (8) | 3 (89) | 2·85 (1·06-7·69) | 2.28 (0.84-6.24) |

*p<0.01 *^1^*Data available from 2011-2021 only for those with noted drugs/alcohol misuse

^†^calculated directly from the raw data without accounting for any confounders

**Table 4. Main model findings of logistic regression for patients and ex-prisoner patients**

|  | Adjusted OR  (95% CI)  (N=3,648) | Adjusted OR  (95% CI)  Forensic community team and probation service excluded  (N=8,109) |
| --- | --- | --- |
| Constant | 0·001 (0·0003-0·008) | 0·002 (0·001-0·004) |
| Sociodemographic characteristics |  |  |
| Age |  |  |
| Under 25 | (reference group) | (reference group) |
| 25-44 | 2.52 (0.90-7·09) | 2.77 (1.54-4.98)* |
| 45-64 | 2.32 (0·77-7·01) | 2.31 (1.22-4.40) |
| 65 and over | 2.81 (0·62-12·76) | 1.33 (0.44-3.98) |
| Unemployed | 1·96 (1·13-3·42) | 1.68 (1.21-2.34) |
| Living alone | 1.47 (0.83-2.60) | 1.05 (0.75-1.48) |
| Unmarried | 0.55 (0.28-1.09) | 1.34 (0.86-2.10) |
| Homeless | 3.14 (0.84-11.80) | 1.30 (0.52-3.26) |
| Primary psychiatric diagnosis |  |  |
| Affective disorder | 0.33 (0.15-0.71) | 0.33 (0.21-0.54)* |
| Personality disorder | 1.27 (0.67-2.41) | 1.37 (0.93-2.01) |
| Drug dependence/misuse | 3·75 (1·73-8·12)* | 3.41 (2.08-5.57)* |
| Other diagnosis | 0.52 (0.20-1.39) | 0.44 (0.22-0.89) |
| Contact with services |  |  |
| Forensic community teams | 43.32 (4·75-394·90)* | - |
| Probation service | 25·82 (9·93-67·14)* | - |
| Lifetime history of alcohol misuse | 1.74 (1.33-2.27)* | 2.02 (1.26-3.23) |
| Lifetime history of drug misuse | 2.57 (1·28-5·17) | 2.75 (1.73-4.36)* |
| Recent (<3 months) alcohol misuse | 0.63 (0.33-1.20) | 1.13 (0.73-1.74) |
| Recent (<3 months) drug misuse | 1.27 (0.61-2.64) | 1.12 (0.72-1.75) |
| Evidence of increased substance use at last contact | 1.78 (1.00-3.16) | 1.18 (0.82-1.69) |
| Deprivation quintiles |  |  |
| 1 | 3.74 (1.10-12.65) | 2.74 (1.39-5.43) |
| 2 | 2.72 (0.78-9.40) | 2.38 (1.19-4.76) |
| 3 | 1.50 (0.38-5.87) | 1.18 (0.54-2.59) |
| 4 | 1.41(0.34-5.77) | 1.34 (0.60-3.00) |
| 5 | (reference group) | (reference group) |
| Suicide methods |  |  |
| Self-poisoning | 1.55 (0.96-2.49) | 1.18 (0.88-1.60) |
