# Supplementary material for: An examination of former prisoners’ mental health problems before death by suicide over a 21-year period (2001–2021)
Source: BJPsych Open. 2025 Jun 23;11(4):e124. doi: 10.1192/bjo.2025.71 (PMC12188233; doi:10.1192/bjo.2025.71)
Supplement: Baird et al. supplementary material 3 — Baird et al. supplementary material [file S2056472425000717sup003.pdf]

THE NATIONAL CONFIDENTIAL INQUIRY INTO SUICIDE AND SAFETY IN MENTAL HEALTH

Address: Professor Sir Louis Appleby, PO Box 86, Manchester, M20 2EF Tel: 0161 275 0700/0701

Suicide Questionnaire

Version: 04/2023

If other mental health professionals have been involved in the management of this patient, please consult them before completing this questionnaire

	Initials	Date
Checked by		
Inputted by		
Counterchecked by		
FOR OFFICE USE ONLY		

Director: Professor Sir Louis Appleby

Work commissioned by The Healthcare Quality Improvement Partnership (HQIP)

SAMPLE

The National Confidential Inquiry will protect the confidentiality of this form but cannot do so for any copies. You are therefore advised not to keep a copy of this questionnaire once it is completed.

SAMPLE

SECTION 1: Priority patients

The National Confidential Inquiry is particularly interested in suicides that occur in the circumstances described below. Please answer the following questions about the patient:

1.1 Was he/she a psychiatric in-patient at the time of suicide (including patients on leave)? ☐

No = 0 Yes = 1

1.2 Did he/she die within 3 calendar months of discharge from psychiatric in-patient care? ☐

No = 0 Yes = 1 Not applicable - in-patient at time of death = 7

1.3 Was he/she under the care of a crisis team* at the time of suicide? ☐

No = 0 Yes = 1

* By crisis team care we mean patients receiving home-based acute care as an alternative to an in-patient admission or to facilitate step-down care, e.g. allowing early discharge. Frequently these are called crisis resolution and home treatment (CRHT) teams but other names are also used.

SAMPLE

SECTION 2: Demographic information (at the time of death)

2.1 Age in years

2.2 Sex

M = 1 F = 2

2.3 Civil Status

1 Divorced/separated
2 Married/co-habiting
3 Single
4 Widowed
5 Same sex marriage/civil partnership

2.4 Employment Status

01 In paid employment (including part-time and self-employed)
02 Unemployed
03 Housewife/husband
04 Full-time student
05 Long-term sick
06 Retired
07 Apprenticeship/training scheme
08 Disability benefit/long-term disability allowance
88 Other (please specify)……………………………………………………..

2.5 Ethnicity/Nationality (based on ONS categories)

Black/African/Caribbean/Black British
01 Black African
02 Black Caribbean
03 Any other Black/African/Caribbean background (please specify)………..
Asian/Asian British
04 South Asian (please specify either 5, 6 or 7 if known)
05 Indian
06 Pakistani
07 Bangladeshi
08 Chinese
09 Any other Asian background (please specify)…………………………………………
White
10 White British
11 Irish
12 Gypsy or Irish traveller
13 White East European
14 Any other White background (please specify)…………………………………..

SAMPLE

Enter what you consider to be the most accurate answer in the box on the right-hand side. If not known, enter 9 if not otherwise specified.

Other

15 Mixed/multiple ethnic group
16 Arab/Middle Eastern
88 Other (please specify)…………………………………………………………
99 Not known

2.6 To your knowledge what was the patient's sexual orientation?

1 Heterosexual
2 Lesbian or gay
3 Bisexual
8 Other (please specify)…………………………………………………………

2.7 Did the patient identify as trans (e.g. transgender, non-binary) group?

No = 0 Yes = 1

2.8 Was the patient:

a) **seeking permission to stay in the UK?** (e.g. asylum seeker, refugee; visa had expired and individual seeking to continue living in the UK)

No = 0 Yes = 1

b) **resident in the UK for less than 5 years?**

No = 0 Yes = 1

2.9 Accommodation at the time of suicide (for in-patients, give accommodation prior to admission)

01 Homeless/no fixed abode
02 Bed & breakfast (long-term)
03 Hostel (supervised or unsupervised)/local authority accommodation
04 Secure children's home/secure training centre
05 House or flat
06 Nursing/care home
07 Prison/Young Offender Institution
08 Immigration Removal Centre/Short-term Holding Facility
09 Sheltered/supported accommodation
88 Other (please specify) ……………………………………………….
99 Not known

SAMPLE

Enter what you consider to be the most accurate answer in the box on the right-hand side. If not known, enter 9 if not otherwise specified.

2.10 Living Circumstances

1 Alone
2 With parent(s)
3 With spouse/partner (with or without children)
4 With child(ren) only (aged under 18)
5 Other shared (e.g. friends)
6 Foster family
7 Prison/Young Offender Institution
8 Other (please specify)………………………………………………

2.11 Was the patient providing care for anyone else in the home?

No = 0 Yes = 1

2.12 Was the patient registered with a GP?

No = 0 Yes = 1

2.13 Was the person a former member of the Armed Forces?

0 No
1 Yes, less than a year ago
2 Yes, between a year and 5 years ago
3 Yes, more than 5 years ago
4 Yes, but not known when

SAMPLE

Enter what you consider to be the most accurate answer in the box on the right-hand side. If not known, enter 9 if not otherwise specified.

SECTION 3: Psychosocial history

3.1 Primary psychiatric diagnosis

01 Schizophrenia or other primary psychotic disorders
02 Drug-induced psychotic disorder
03 Bipolar affective disorder
04 Depressive disorder
05 Anxiety disorder/phobia/panic disorder/OCD
06 PTSD
07 Eating disorder
08 Dementia
09 Alcohol dependence/misuse
10 Drug dependence/misuse
11 Personality disorder
12 Adjustment disorder
13 Organic disorder
14 Learning disability
15 Autism spectrum disorder
16 ADHD
17 Conduct-dissocial disorder
18 Somatoform/somatisation disorder
19 Mental disorder present but not able to specify
20 No information available/information lacking
77 No mental disorder (i.e. not 01 to 19 or 88)
88 Other (please specify) …………………………………………………………..
99 Not known

3.2 Secondary Diagnosis (coding as above)

1) 2) 3) 4)

3.3 If the patient was diagnosed with personality disorder, what type was it?

1 Antisocial
2 Emotionally unstable/Borderline
3 Other (please specify)…………………………………………………………..
7 Not applicable

3.4 If the patient had psychosis, was the most recent episode a first episode psychosis?

No = 0 Yes = 1 Not applicable = 7

Enter what you consider to be the most accurate answer in the box on the right-hand side. If not known, enter 9 if not otherwise specified.

SAMPLE

3.5 If the patient had an autism spectrum disorder, had this been diagnosed before contact with mental health services?

No = 0 Yes = 1 Not applicable = 7

3.6 Duration of mental illness (since clear onset of disorder coded under 3.1)

1 Less than 3 months
2 3-12 months (but less than a year)
3 1-5 years
4 More than 5 years
7 No mental disorder

3.7 When was the first contact with mental health services?

1 Less than 3 months ago
2 3-12 months ago (but less than a year)
3 1-5 years ago
4 More than 5 years ago

Physical illness

The following questions ask about any major physical illness the patient had at the time of death.

3.8 Did the patient have a major physical illness at the time of death? (include conditions even if well controlled by treatment)

No = 0 Yes = 1 **[If no , please go to question 3.9]**

3.9 If yes, did the patient have any of the following major physical illnesses?

No = 0 Yes = 1

Cancer

Cardiovascular disease, including cerebrovascular disease

Chronic pain

Diabetes

Digestive system disease

Disease of the nervous system

SAMPLE

Endocrine & metabolic disease

Impaired mobility

Musculoskeletal disease

Respiratory disease

Other (please specify)……………………………………………………………………………..

Specific physical diagnosis (optional)…………………………………………………………..

3.10 Did the patient have any hearing impairments at the time of death?

No = 0 Yes = 1

3.11 If yes, was the patient in contact with any specialist mental health support services relating to this hearing impairment?

No = 0 Yes = 1 Not applicable (patient had no hearing impairment) = 7

Patients aged under 25

The following questions are for patients who were aged under 25 at the time of death. If the patient was not aged under 25, please go to question 3.13.

3.12 Was there a history of Local Authority Care?
(e.g. in a children's home or foster care)

No = 0 Yes = 1

3.13 Did the patient previously have a childhood psychiatric diagnosis?

No = 0 Yes = 1

3.14 If the answer to 3.10 is yes, what was the primary diagnosis?

1 ADHD
2 Conduct disorder
3 Autism spectrum disorder
4 Emotional disorder, e.g. depression, anxiety, etc
5 Psychosis
8 Other (please specify)…………………………………………………………

SAMPLE

3.15 Had the patient been under the care of the following agencies as a child/minor:

No = 0 Yes = 1

Young Offender Institution

Child protection

Youth justice team

Secure care under Local Authority services
(e.g. secure children's home/secure treatment centre)

Child and adolescent mental health services

Other (please specify)……………………………………………………………………………

Lifetime history

The following questions ask about the lifetime experience of the patient.

Did the patient have a history of the following at any time?

No = 0 Yes = 1

3.16 Admission to a high security hospital/medium secure unit

3.17 Treatment by specialist military mental health services

3.18 Being in prison (including being a remand prisoner)

3.19 Exposure to suicide (family, partner, friends)

3.20 Self-harm

3.21 Alcohol misuse

3.22 Drug misuse

3.23 If there was a history of drug misuse, which substances were misused?
(Base your answer on frequency and potential harmfulness)

No = 0 Yes = 1 Not applicable = 7

Heroin/other opiates

Stimulants (e.g. amphetamines, LSD, mushrooms, crack/cocaine, ecstasy)

SAMPLE

Ketamine

Benzodiazepines (other than as prescribed)

Cannabis

Skunk (or other potent forms of cannabis)

New psychoactive substances (often called 'legal highs')

Other (please specify)………………………………………………………

3.24 Childhood abuse

No = 0 Yes = 1

Physical

Psychological or emotional

Sexual

3.25 Domestic/intimate partner violence, i.e. as a victim

No = 0 Yes = 1

Sexual assault

Physical assault

Psychological or emotional abuse

3.26 Violence as a perpetrator (includes serious threat)

No = 0 Yes = 1

Sexual assault

Physical assault

Psychological or emotional abuse

SAMPLE

Recent experiences

The following section is on life events or stressors experienced in the **3 months** **before the suicide.**

Did the patient recently experience any of the following?

No = 0 Yes = 1

Social:

3.27 Serious financial difficulties

3.28 If yes, did these financial difficulties relate to any of the following?

No = 0 Yes = 1 Not applicable = 7

Debt (e.g. loans, credit or store cards)

Mortgage/rent arrears

Loss of welfare benefits/disability benefits

Instability or loss of job

Loss of housing

Other (please specify)……………………………………………………………………………

3.29 Workplace stressors (e.g. intimidation or bullying)

3.30 Assessment for, or change in, welfare benefits such as Universal Credit, Personal Independence Payment (PIP), Adult Disability Payment

3.31 Gambling

3.32 Alcohol misuse

3.33 Drug misuse

3.34 If there was recent drug misuse, which substances were misused?
(Base your answer on frequency and potential harmfulness)

No = 0 Yes = 1 Not applicable = 7

Heroin/other opiates

Stimulants (e.g. amphetamines, LSD, mushrooms, crack/cocaine, ecstasy)

SAMPLE

Benzodiazepines (other than as prescribed)

Cannabis

Skunk (or other potent forms of cannabis)

Ketamine

New psychoactive substances (often called 'legal highs')

Other (please specify)…………………………………………………………………………………….

Criminality and violence:

3.35 Criminal charges

3.36 Victim of:

Crime

Hate crime

Stalking

Intimidation

3.37 Domestic/intimate partner violence, i.e. as a victim

Sexual assault

Physical assault

Psychological or emotional abuse

3.38 Violence as a perpetrator (includes serious threat)

Sexual assault

Physical assault

Psychological or emotional abuse

Emotional and physiological:

3.39 Bereavement

Enter what you consider to be the most accurate answer in the box on the right-hand side. If not known, enter 9 if not otherwise specified.

SAMPLE

3.40 Menopause

3.41 Fertility problems or concerns

3.42 Insomnia

3.43 Self-harm

If there was no recent self-harm, please go to question 3.48

3.44 When did this episode of self-harm occur before the suicide?

1 Less than 1 week
2 More than a week but less than a month
3 Between 1 and 3 months
7 Not applicable – no recent self-harm

3.45 What method of self-harm was used in this recent episode?

…………………………………………………………………………………………………..

3.46 Did this episode of self-harm lead to contact with services (including the Emergency Department)?

No = 0 Yes = 1

3.47 If yes, what was the result?

1 Not assessed
2 Assessment only – no follow-up
3 Referral to a mental health team for further follow-up
4 Referral to another service for follow-up
5 Admission to a psychiatric bed
7 Not applicable
8 Other (please specify)…………………………………………………………………….

3.48 Any other recent life events or stressors (please specify)…………………………

………

………

………

………

SAMPLE

Coronavirus (COVID-19) outbreak

3.49 Was the COVID-19 outbreak, or any restrictions, implicated as contributing to the suicide?

No = 0 Yes = 1

3.50 If yes, did any of the following occur due to the COVID-19 outbreak?

No = 0 Yes = 1 Not applicable = 7

Loss of job

Other financial stressors

Physically ill with the virus

Bereavement

Disruption to formal support from mental health services

Isolation or limitations to daily life

Victim of domestic violence

Increased use of alcohol

Other (please specify)…………………………………………………………………………………

………

SAMPLE

SECTION 4: DETAILS OF SUICIDE

Before sending you this form, we have usually been informed that the death has been classified as suicide or undetermined.

4.1 **Method** (if more than one, please give direct cause)

01 Self-poisoning
02 Strangulation
03 Hanging
04 Drowning
05 Firearms
06 Cutting or stabbing
07 Jumping from a height/multiple injuries
08 Jumping/lying before a train
09 Jumping/lying before any other vehicle
10 Burning
11 Electrocution
12 Suffocation/asphyxiation
13 Inhalation of gases (please specify)

………
88 Other (please specify)

………
99 Not known

4.2 **If self-poisoning, specify substance** (if more than one substance, select most likely cause of death). **If not self-poisoning, please go directly to question 4.5.**

01 Antipsychotic drugs
02 Tricyclic anti-depressant
03 SSRI/SNRI anti-depressant
04 Lithium/Mood stabiliser
05 Other anti-depressant
06 Benzodiazepine/Hypnotic
07 Paracetamol
08 Paracetamol/opiate compound
09 Other analgesic
10 Opiate (heroin, methadone, etc)
11 Insulin
12 Other poisons (e.g. weed killer)
88 Other substance (please specify)

………..
99 Not known

SAMPLE

Enter what you consider to be the most accurate answer in the box on the right-hand side. If not known, enter 9 if not otherwise specified.

4.3 If the substance in question 4.2 was an opiate, what type was it?

1 Heroin/morphine
2 Methadone
3 Codeine
4 Tramadol
7 Not applicable - substance was not an opiate
8 Other (please specify)…………………………………………………

……………………………………………………………………………………

4.4 If the substance in question 4.2 was an opiate or a paracetamol/opiate compound, how was it obtained?

1 Prescribed for the patient for treatment of pain
2 Prescribed for the patient for treatment of drug misuse
3 Prescribed for someone else
4 Illicitly
5 Not prescribed, i.e. over the counter
7 Not applicable - substance was not an opiate or paracetamol/opiate compound
8 Other (please specify)……………………………………………………

……………………………………………………………………………………

4.5 Did the suicide occur in the following circumstances?

No = 0 Yes = 1

Homicide followed by suicide

Died in a suicide pact

4.6 Was there evidence of suicide-related internet use?

No = 0 Yes = 1

Obtained information (e.g. method details) on how to die by suicide (please specify source of information)

……………………………………………………………………………………………

Visited websites that may have discussed/encouraged suicide, including chat rooms

Communicated suicidal ideas/intent online

Communicated suicidal ideas/intent via social media (e.g. Twitter, Instagram, Facebook)

Experienced online bullying

Other (please specify)…………………………………………………………………

Enter what you consider to be the most accurate answer in the box on the right-hand side. If not known, enter 9 if not otherwise specified.

SAMPLE

4.7 Did the suicide occur in a woman who was pregnant or post-natal? ☐

0 No
1 Yes, woman was pregnant
2 Yes, suicide was less than one year after childbirth

4.8 Did the suicide occur in any of these settings? ☐

01 Home
02 Hospital ward
03 Multi-storey car park
04 Bridge
05 Coastal location
06 River location
07 Railway location
08 Road/highway location
09 Park/woods
88 Other public place (please specify)

……………………………………………………………………………………………………….

4.9 To your knowledge, was the location of suicide a place of emotional significance? ☐

0 No
1 A place of death of a family member or friend
2 A place someone else they knew died by suicide
3 Any other emotionally significant place (please specify)

……………………………………………………………………………………………………….

4.10 To your knowledge, did the suicide occur on or near an anniversary or a significant date? ☐

0 No
1 The patient's birthday
2 Anniversary of a death of a family member or friend
3 Any other significant date (please specify)

……………………………………………………………………………………………………….

4.11 Was the suicide thought to be part of a cluster of suicides? ☐

No = 0 Yes = 1

Enter what you consider to be the most accurate answer in the box on the right-hand side. If not known, enter 9 if not otherwise specified.

SAMPLE

SECTION 5: In-patient suicides

Complete this section only if the patient was a **psychiatric in-patient** at the time of suicide (including patients on leave). Otherwise, go to Section 6 (page 16).

5.1 Date of admission to the in-patient unit

Day **Month** **Year**

5.2 Did the suicide occur within 7 days of admission?

No = 0 Yes = 1

5.3 Please provide the time of death (if unknown please put best estimate using 24-hour clock)

5.4 Was the ward where the patient died within the local in-patient unit?

No = 0 Yes = 1

5.5 Type of ward (if patient was on leave, where they were prior to leave)

1 General psychiatry open ward
2 Psychiatric intensive care ward
3 Low/medium secure unit or high secure hospital
4 Rehabilitation unit
5 CAMHS ward (including forensic ward)
6 Eating disorders ward/unit
7 Older person's unit
8 Other (please specify) ………………………………………………………………….

5.6 Patient's legal status at the time of suicide

1 Informal/voluntary
2 Detained for assessment
(section 2 in England & Wales; forms 7, 8 or 9 in Northern Ireland)
3 Detained for treatment
(section 3 in England & Wales; forms 10, 11 or 12 in Northern Ireland)
4 Detained under short term emergency section or under doctors' or nurses' holding powers
(section 4 or 5 in England & Wales; forms 5 or 6 in Northern Ireland)
5 Detained under forensic section (Part 3 of MHA) or similar
8 Other (please specify) ………………………………………………………………….

Enter what you consider to be the most accurate answer in the box on the right-hand side. If not known, enter 9 if not otherwise specified.

SAMPLE

5.7 Did any of the following occur at the last admission?

No = 0 Yes = 1

Manual restraint

Seclusion

Urgent intramuscular (IM) or intravenous (IV) medication

5.8 Patient's observation status at the time of suicide

1 Constant (observation within eyesight or within arm's length)
2 Intermittent (observation every 15-30 minutes)
3 General observation
7 Not applicable - patient was on leave
8 Other (please specify)……………………………………………………………….

5.9 Were there particular problems in observing this patient on the ward because of any of the following?

No = 0 Yes = 1

Ward design

Staff shortages

Staff were busy, e.g. with other patients, handover

Other (please specify)……………………………………………………………………………….

5.10 Where did the suicide take place?

1 On the ward
2 In hospital grounds (not on the ward)
3 Off hospital grounds
8 Other (please specify) ………………………………………………………………..

5.11 If the patient was off the ward, , to what extent had leave been granted at the time of suicide?

1 Patient was on agreed leave
2 Patient was off the ward with staff agreement
3 Patient was off the ward without staff agreement
4 Patient was off the ward and had not returned from agreed leave
7 Not applicable - patient was on the ward at the time of suicide
8 Other (please specify) ……………………………………………………………..

SAMPLE

5.12 If the patient was off the ward <u>with staff agreement</u>, was the patient:

No = 0 Yes = 1 Not applicable = 7

On agreed leave as a step towards planning discharge

On escorted leave (with a member of staff or family)

5.13 If the patient was off the ward <u>without staff agreement</u>, how did they leave?

No = 0 Yes = 1 Not applicable = 7

Through the main unit door

By scaling a barrier (e.g. perimeter fence)

Other (please specify) …………………………………………………………………………………

5.14 If the suicide took place on the ward, what was the location of the suicide?

1 Shared room or dormitory
2 Single bedroom
3 Toilet/bathroom
4 En-suite bathroom
7 Not applicable - suicide occurred off the ward
8 Other (please specify)…………………………………………………

…………………………………………………………………………………

5.15 If the suicide occurred on the ward by hanging/strangulation or asphyxiation:

a) What did the patient use in dying by this method?

1 Sheet, towel etc.
2 Tie
3 Belt
4 Shoelaces
5 Item brought in specifically for purpose (e.g. rope)
6 Plastic bag
7 Not applicable - death was not by hanging/strangulation or asphyxiation on ward
8 Other (please specify)…………………………………………………

SAMPLE

b) What did the patient hang/strangle him/herself from?

01 Bed curtain rail
02 Pipes
03 Hook or handle
04 Door
05 Bed head
06 Window
07 Self-strangulation, i.e. no ligature point
77 Not applicable - death was not by hanging/strangulation on ward
88 Other (please specify)………………………………………………….

c) Did the patient use a low-lying ligature point (i.e. below head height)?

No = 0 Yes = 1 Not applicable – death was not by hanging/strangulation on the ward = 7

SAMPLE

SECTION 6: Community patients (including patients no longer in contact with services)

Complete this section if the patient was living outside hospital at the time of suicide. Questions relating to CTO will only apply to England and Wales.

Last admission

6.1 Nature of last admission to psychiatric in-patient care

0 None **[If none, please go directly to question 6.13]**
1 Informal/voluntary
2 Detained for assessment
(section 2 in England & Wales; forms 7, 8 or 9 in Northern Ireland)
3 Detained for treatment
(section 3 in England & Wales; forms 10, 11 or 12 in Northern Ireland)
4 Detained under short term emergency section or under doctors' or nurses' holding powers
(section 4 or 5 in England & Wales; forms 5 or 6 in Northern Ireland)
5 Detained under forensic section (Part 3 MHA) or similar
6 Under the power of recall of a Community Treatment Order (CTO)
8 Other (please specify) ………………………………………………………………

………………………………………………………………………………………………

6.2 Was the last admission to the local in-patient unit?

No = 0 Yes = 1

6.3 Duration of last admission

1 3 days or less
2 4-7 days
3 More than 1 week but less than 4 weeks
4 Between 4 and 13 weeks
5 More than 13 weeks

6.4 Was this a re-admission within 3 months of a previous discharge from psychiatric in-patient care?

No = 0 Yes = 1

SAMPLE

Enter what you consider to be the most accurate answer in the box on the right-hand side. If not known, enter 9 if not otherwise specified.

Post-discharge and follow-up

6.5 Date of last discharge from psychiatric in-patient care (if patient was discharged in last year)

Day **Month** **Year**

(Note. Please enter **77** if the patient was not discharged in last year or enter **99** if date was unknown)

6.6 When did the suicide occur after discharge?

1 Less than 3 days
2 Between 3 and 6 days
3 7 days or more

6.7 Nature of last discharge from psychiatric in-patient care

1 Planned
2 Discharge following self-harm or breach of ward rules (e.g. drinking, violence)
3 Self-discharge
8 Other (please specify) …………………………………………………………..........

6.8 In your opinion, had the patient been discharged before risk had sufficiently been reduced?

No = 0 Yes = 1

6.9 If yes, in your opinion was this due to any of the following factors?

No = 0 Yes = 1 Not applicable = 7

Pressure on beds

Pressure from the patient or family

Policy locally

Other (please specify) ……………………………………………………...................

6.10 Was the patient being discharged to any of the following unresolved problems?

No = 0 Yes = 1

Housing, financial, employment problems

Poor social support

SAMPLE

Drug/alcohol misuse

Physical ill health

Other (please specify)……………………………………………………………………………

6.11 Following discharge from psychiatric in-patient care, when was the first follow-up contact with a member of the multi-disciplinary team?

0 No follow-up arranged **[If no follow-up, please go to question 6.13]**
1 Within 3 days
2 More than 3 days but within a week
3 A week or more
8 Other (please specify)

……….

6.12 Did the suicide occur before the follow-up appointment took place?

No = 0 Yes = 1

6.13 What was the nature of the first follow-up contact with psychiatric services?

1 Face-to-face
2 Telephone
3 SMS/email
4 Zooms, Teams, Skype (or similar)
8 Other (please specify)…………………………………………………………………………….

Crisis teams/CRHT

If the patient was not under the care of CRHT at the time of death, please go to question 6.15

6.14 How long had the patient been under the care of CRHT services?

1 Less than 24 hours
2 More than 1 day but less than 1 week
3 A week or more

6.15 Did the care plan under CRHT include provision of additional social support at home, e.g. from a relative, friend or neighbour?

No = 0 Yes = 1

SAMPLE

Community Treatment Order

6.16 Was the patient made subject to a Community Treatment Order (CTO) at the time of his/her last discharge?

No = 0 Yes = 1 Not applicable - no previous admission = 7

6.17 Was the patient subject to a CTO at the time of death?

1 Yes
2 No, CTO revoked
7 Not applicable – not subject to CTO or no previous admission

6.18 How long had the patient been under CTO?

1 Less than 6 months
2 Between 6 months and a year
3 A year or more
7 Not applicable – not subject to CTO or no previous admission

Early intervention

6.19 Was the patient seen under Early Intervention services?

No = 0 Yes = 1

6.20 If yes, was the patient seen within two weeks of referral?

No = 0 Yes = 1 Not applicable - patient not seen under EI = 7

Section 136

6.21 Was the patient conveyed to a <u>hospital based</u> place of safety under S136 of the MHA (or Article 130 MHO in Northern Ireland) in the <u>3 months</u> prior to suicide?

No = 0 Yes = 1

6.22 If yes, what was the result?

1 Assessment only - no follow-up
2 Referral to a mental health team for further follow-up
3 Referral to another service for follow-up
4 Admission
7 Not applicable
8 Other (please specify) ……………………………………………………………………..

Enter what you consider to be the most accurate answer in the box on the right-hand side. If not known, enter 9 if not otherwise specified.

SAMPLE

6.23 Was the patient conveyed to a <u>custody based</u> place of safety under S136 of the MHA (or Article 130 MHO in Northern Ireland) in the <u>3 months</u> prior to suicide?

No = 0 Yes = 1

6.24 If yes, what was the result?

1 Assessment only - no follow-up
2 Referral to a mental health team for further follow-up
3 Referral to another service for follow-up
4 Admission
7 Not applicable
8 Other (please specify) ……………………………………………………………………..

Recent contact with services

We have asked you about contact the patient had with a range of specialist services. In addition to these, we would like to know whether there was contact with other teams or services.

6.25 Which other services was the patient under at the time of death?

No = 0 Yes = 1

Acute day hospital

Alcohol services

(Assertive) outreach service

CAMHS

Children's social services (including child protection services)

CMHT

Criminal justice liaison and diversion team/street triage

Drug services

Employment services

Forensic community team

IAPT or Primary Mental Health Support Service (PMHSS) in Wales

SAMPLE

Liaison psychiatry

Maternal/perinatal mental health

Older peoples' mental health services

Probation

Recovery/rehabilitation

Specialist military mental health services

Specialist personality disorder service

Specialist psychotherapy services

Other (please specify)……………………………………………………………………………………

6.26 Was the contact the patient had with services a one-off contact?

No = 0 Yes = 1

6.27 Was there a transfer of care from another trust (health board) in the 12 months prior to suicide

No = 0 Yes = 1

If yes, please specify…………………………………………………………………………………

6.28 Had the patient been subject to an <u>urgent</u> referral to specialist mental health services by a GP in the <u>3 months</u> prior to suicide?

No = 0 Yes = 1

6.29 If yes, what was the result?

1 Assessment only – no follow-up
2 Referral to mental health team but not seen
3 Admission
4 Not yet seen
7 Not applicable
8 Other (please specify) ……………………………………………………………………….

SAMPLE

6.30 Had the patient been subject to a <u>routine</u> referral to specialist mental health services by a GP in the <u>3 months</u> prior to suicide?

No = 0 Yes = 1

6.31 If yes, what was the result?

1 Assessment only – no follow-up
2 Referral to mental health team but not seen
3 Admission
4 Not yet seen
7 Not applicable
8 Other (please specify) ……………………………………………………………………….

6.32 Did any of the following occur when accessing mental health care? (please tick all that apply)

No = 0 Yes = 1

1 A delay in providing mental health care due to the volume of referrals

2 Service was unable to provide the treatment requested

3 Patient was not accepted as the clinical problem was not severe enough

4 Patient was not suitable for care/treatment due to comorbid problems (e.g. self-harm, substance misuse)

5 Patient was on a waiting list for admission

6.33 Had there been a transition from CAMHS to adult services in the previous year?

No = 0 Yes = 1

Loss of contact with services

6.34 Did the patient miss their last appointment (with any member of the mental health team in a clinic or in the community)?

No = 0 Yes = 1

SAMPLE

6.35 Following the missed appointment, what action was taken?

No = 0 Yes = 1 Patient did not miss final appointment = 7

Patient discharged from follow-up

Further appointment/letter sent

Telephone call to patient to arrange follow-up

Professional home visit (face-to-face)

GP informed

Contact between mental health team and patient's family

SAMPLE

SECTION 7: Treatment (<u>all patients</u>)

The following questions refer to the treatment that the patient was receiving at the time of death.

7.1 Which of the following interventions was the patient receiving at the time of death?

No = 0 Yes = 1

Drug treatment

Antipsychotics:

Oral

Depot

Antidepressants:

Tricyclics

SSRI/SNRI and related

Lithium/mood stabilisers

Other antidepressants

Benzodiazepines

Other psychotropic drugs

Other drug treatment (please specify)………………………………………………...

Psychological treatment (a course of CBT, family therapy, IPT, group therapy)

(please specify) ……………………………………………………………………………………….

Educational/employment support

(please specify) ……………………………………………………………………………………….

7.2 Was he/she refusing to take medication as prescribed (e.g. non-adherent) <u>in the month</u> before death?

No = 0 Yes = 1

(<u>Note:</u> Enter 0 if no medication was prescribed)

SAMPLE

7.3 Did the patient complain of distressing psychotropic drug side-effects?

No = 0 Yes = 1 Not prescribed medication = 7

7.4 If yes, what type of side effects did the patient complain of?

No = 0 Yes = 1 No side effects/not prescribed medication = 7

Weight gain

Extrapyramidal symptoms

Sedation

Arousal/agitation/insomnia

Sexual dysfunction

Other (please specify) …………………………………………………………………

7.5 What was the main reason for not taking medication as prescribed?

1 Side effects
2 Lack of insight into illness
3 Dependence (e.g. persistent benzodiazepine use against medical advice)
4 Patient found no positive effect from medication
5 Stigma attached to taking medication
6 Not applicable - patient not prescribed medication
7 Not applicable - patient was taking medication as prescribed
8 Other (please specify) …………………………………………………………

SAMPLE

SECTION 8: Last contact (all patients)

The following questions refer to the **last formal contact or appointment with a member of the mental health team** before suicide (i.e. telephone or face-to-face contact). In the case of in-patients this refers to the **last consultation with a member of clinical staff.**

8.1 How long before the suicide did the last contact occur?

1 Less than 24 hours
2 1-7 days
3 More than 1 week to 4 weeks
4 More than 4 weeks to 13 weeks
5 More than 13 weeks

8.2 What was the nature of the last contact?

1 Face-to-face
2 Telephone
3 SMS or email
4 Zoom, Teams, Skype (or similar)
8 Other (please specify)………………………………………………………

8.3 What was the reason for this last contact?

1 Routine/non-urgent
2 Urgent request by patient
3 Urgent request by family
4 Urgent request by professional
5 Formal police referral (e.g. Section 136 in England & Wales, Article 130 in N.I.)
6 Assessment after self-harm
7 Request for self-discharge (in-patient)
8 Other (please specify) ………………………………………………......

8.4 Where did this last contact take place?

01 Patient's home
02 Community/GP clinic
03 Emergency department
04 Mental Health Unit (including outpatients and day hospitals)
05 Psychiatric in-patient ward
06 Telephone/video call contact
07 Medical ward
08 Criminal justice setting
88 Other (please specify)……………………………………………………..
99 Not known

Enter what you consider to be the most accurate answer in the box on the right-hand side. If not known, enter 9 if not otherwise specified.

SAMPLE

8.5 Was there clear evidence of any of the following at last contact?

No = 0 Yes = 1

Deterioration in mental state

Increased use of alcohol/drugs

Decrease in social support

Increasing suicidal ideas or self-harm

Risk assessment

The following questions refer to the assessment of suicide risk.

8.6 How high was the <u>long term</u> risk thought to be, at last contact?

1 No risk
2 Low
3 Moderate
4 High
5 Risk not considered
6 Risk not categorised in this way

8.7 How high was the <u>immediate</u> risk thought to be, at last contact?

1 No risk
2 Low
3 Moderate
4 High
5 Risk not considered
6 Risk not categorised in this way

8.8 How was the risk assessed?

1 Clinical assessment
2 Local risk tool
3 Standardised risk tool
7 Not applicable – risk not considered
8 Other (please specify)……………………………………………………………………………….

SAMPLE

8.9 If the immediate risk was viewed as moderate or high, was the management plan changed after the assessment?

No = 0 Yes = 1 Not applicable = 7

8.10 If the management plan was not changed, was this due to any of the following factors?

No =0 Yes = 1 Not applicable = 7

Patient was viewed as having mental capacity to make safety decisions

Patient refused additional input

The family was able to provide additional support

Other (please specify)……………………………………………………………………………..

SAMPLE

SECTION 9: Your view on prevention

Which of the following would have made the suicide significantly less likely at that time?

No = 0 Yes = 1

9.1 Better supervision of junior/inexperienced staff

9.2 Increased staffing

9.3 Better staff training in risk assessment

9.4 Closer supervision of patient

9.5 Closer working with GP

9.6 Use of mental health legislation

(please specify)……………………………………………………………………………………

9.7 Better communication between teams

9.8 Less frequent use of agency/locum staff

9.9 Closer contact with patient's family

9.10 Better out of hours care

9.11 Greater availability of psychiatric beds

9.12 Decrease in case loads

9.13 Better crisis facilities

9.14 Availability of dual diagnosis, alcohol or drug services

9.15 Increased access or lower waiting times for psychological therapies

Enter what you consider to be the most accurate answer in the box on the right-hand side. If not known, enter 9 i[illegible] not otherwise specified.

SAMPLE

9.16 Patient taking medication in line with treatment plan (e.g. adherence with treatment)

9.17 Other (please specify)……………………………………………………………………………

………

SECTION 10: Case review

10.1 Please use this section to give us any additional information that has not already been covered

………

………

………

10.2 Can you give examples of good practice in your service that other services might adopt?

………

………

………

The following questions relate to the time period following the suicide:

10.3 Has there been a review or investigation of the case <u>following</u> the patient's death?

No = 0 Yes = 1

10.4 Did the relatives/carers of the patient take part in the review process?

No = 0 Yes = 1 Not applicable (no review) = 7

10.5 Did the relatives/carers of the patient receive any formal support <u>following his/her death?</u>

No = 0 Yes = 1

If yes, please specify…………………………………………………………………………

10.6 In your opinion, were positive changes made to mental health care as a result of the review or internal investigation?

Enter what you consider to be the most accurate answer in the box on the right-hand side. If not known, enter 9 if not otherwise specified.

SAMPLE

0 = No
1 = Yes, within the team
2 = Yes, within the wider trust/health board
7 = Not applicable

If yes, please specify…………………………………………………………………………….

SECTION 11: Your details

11.1 Were you the clinician responsible for the patient's care at any point during the year before death?

No = 0 Yes = 1

11.2 Did you know the patient personally?

No = 0 Yes = 1

11.3 Completed by (Name): …………………………………………………………….

11.4 Signature: …………………………………………………………….

11.5 Job title: …………………………………………………………….

11.6 Contact telephone number: …………………………………………………………….

Thank you for completing this questionnaire.
Please return to Professor Sir Louis Appleby, PO Box 86, Manchester, M20 2EF

FOR OFFICE USE ONLY:

11.7 Country in which patient was treated:

1	England	2	Wales
3	Scotland	4	Northern Ireland

Enter what you consider to be the most accurate answer in the box on the right-hand side. If not known, enter 9 if not otherwise specified.

SAMPLE

© 2023 Healthcare Quality Improvement Partnership (HQIP)

SAMPLE

Enter what you consider to be the most accurate answer in the box on the right-hand side. If not known, enter 9 if not otherwise specified.
